# Author Correction: Genotoxic stress triggers Scd6-dependent regulation of translation to modulate the DNA damage response

**DOI:** 10.1038/s44319-025-00481-x

**Published:** 2025-05-20

**Authors:** Gayatri Mohanan, Raju Roy, Hélène Malka-Mahieu, Swati Lamba, Lucilla Fabbri, Sidhant Kalia, Anusmita Biswas, Sylvain Martineau, Céline M Labbé, Stéphan Vagner, Purusharth I Rajyaguru

**Affiliations:** 1 Department of Biochemistry, Indian Institute of Science, Bangalore, Karnataka 560012 India; 2 Institut Curie, PSL Research University, CNRS UMR3348, INSERM U1278, F-91405 Orsay, France; 3 Université Paris Sud, Université Paris-Saclay, CNRS UMR3348, INSERM U1278, F-91405 Orsay, France; 4 Present Address: University of Pennsylvania, Philadelphia, PA USA

**Correction to:** *EMBO Reports* (2025). 10.1038/s44319-025-00443-3 | Published online 24 April 2025

The authors detected a textual error in Figure 5C

**Figure:**
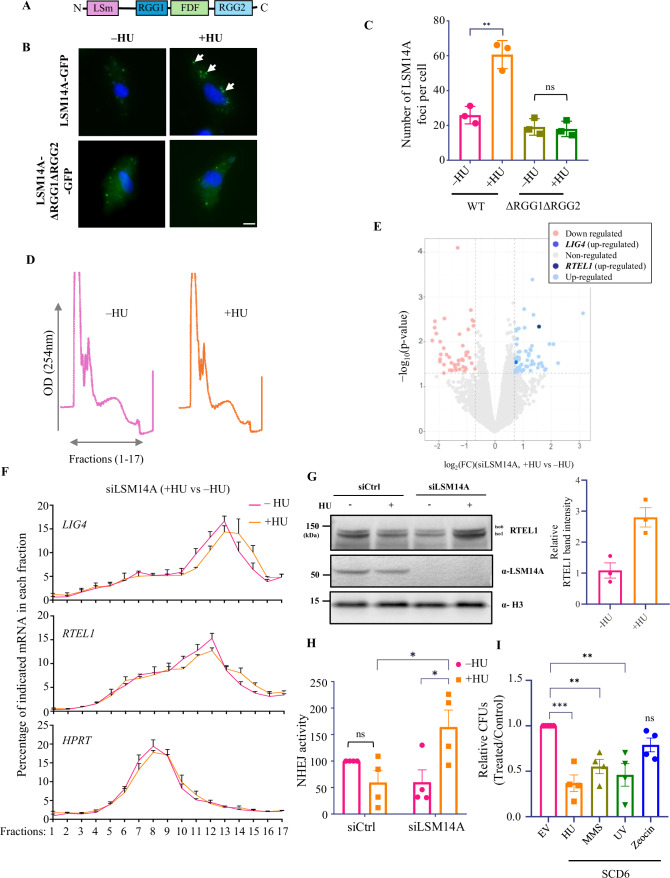
Figure 5C is corrected.

