# Supplementary figures and images for: Genotoxic stress triggers Scd6-dependent regulation of translation to modulate the DNA damage response
Source: EMBO Rep. 2025 Apr 24;26(10):2715–39. doi: 10.1038/s44319-025-00443-3 (PMC12116771; doi:10.1038/s44319-025-00443-3)

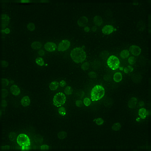

Supplement: Supplementary file 4 — Source data Fig. 1 [file 44319_2025_443_MOESM4_ESM.zip › Figure 1/1A_microscopy raw data/Cisplatin.tif]

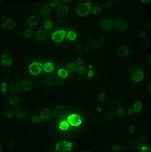

Supplement: Supplementary file 4 — Source data Fig. 1 [file 44319_2025_443_MOESM4_ESM.zip › Figure 1/1A_microscopy raw data/Control.tif]

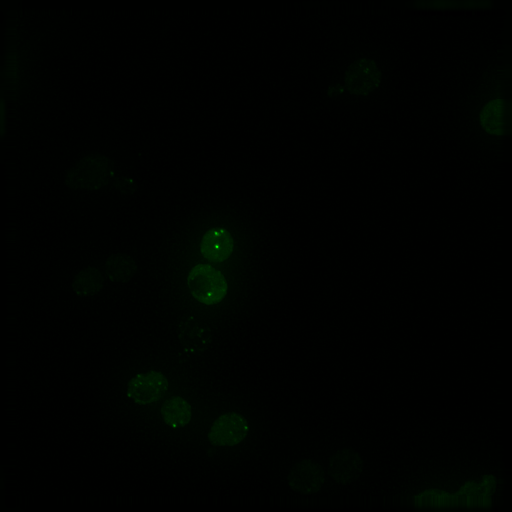

Supplement: Supplementary file 4 — Source data Fig. 1 [file 44319_2025_443_MOESM4_ESM.zip › Figure 1/1A_microscopy raw data/HU.tif]

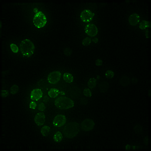

Supplement: Supplementary file 4 — Source data Fig. 1 [file 44319_2025_443_MOESM4_ESM.zip › Figure 1/1A_microscopy raw data/MMS.tif]

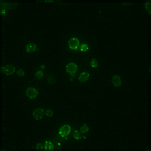

Supplement: Supplementary file 4 — Source data Fig. 1 [file 44319_2025_443_MOESM4_ESM.zip › Figure 1/1A_microscopy raw data/UV.tif]

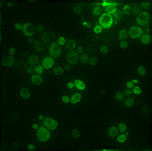

Supplement: Supplementary file 4 — Source data Fig. 1 [file 44319_2025_443_MOESM4_ESM.zip › Figure 1/1A_microscopy raw data/Zeocin.tif]

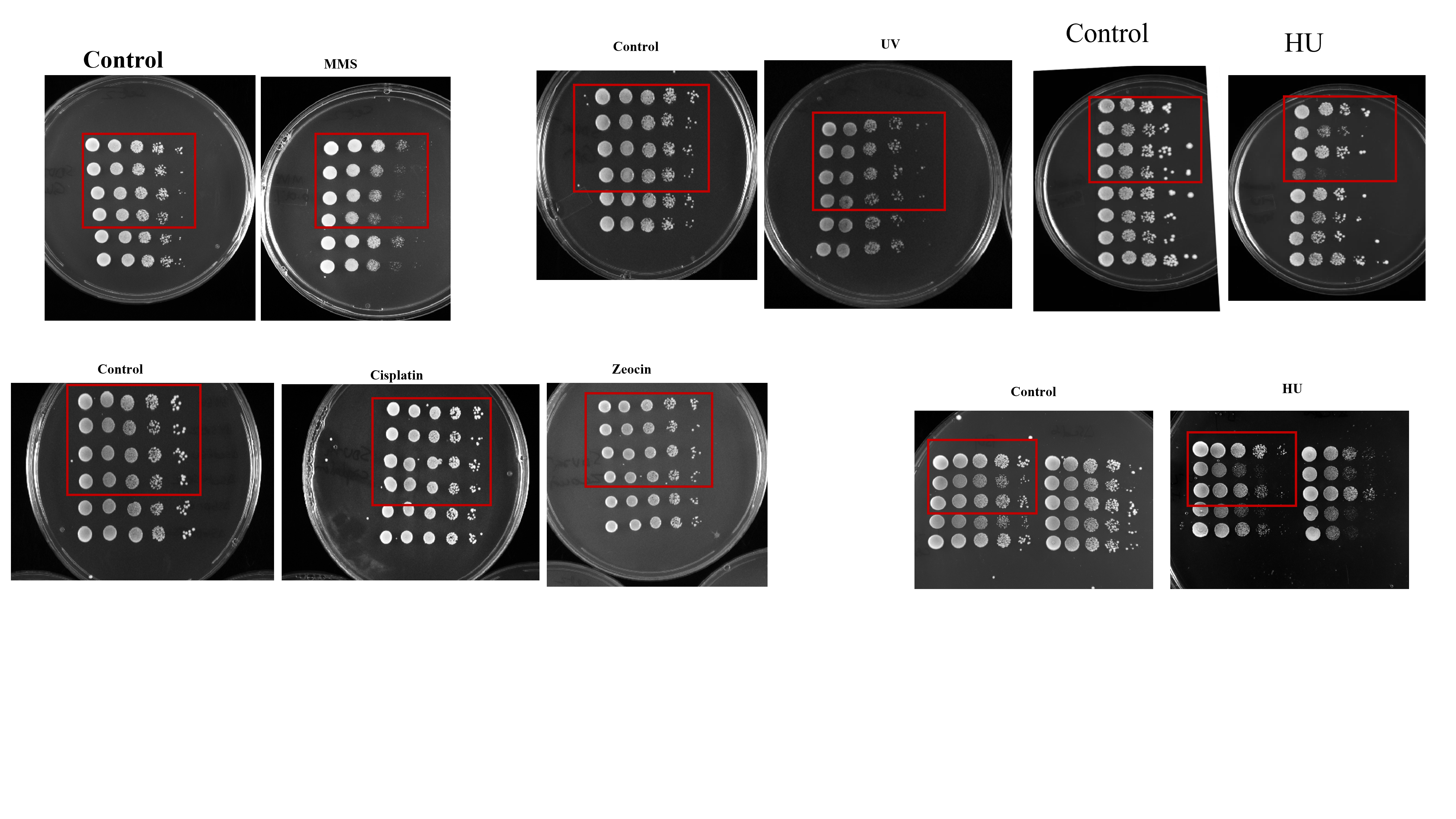

Supplement: Supplementary file 5 — Source data Fig. 2 [file 44319_2025_443_MOESM5_ESM.zip › Figure 2/2A 2B and 2C/Growth assay plates.tif]

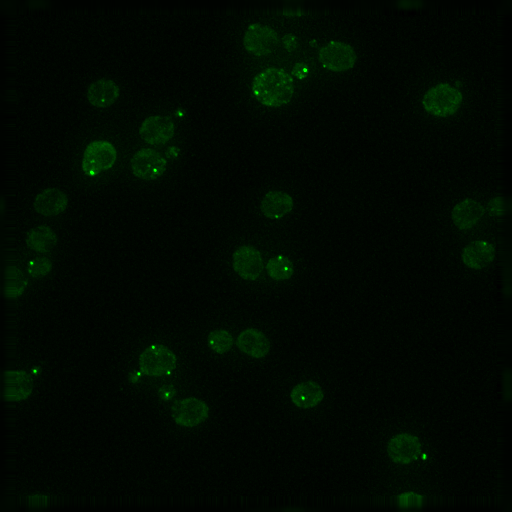

Supplement: Supplementary file 5 — Source data Fig. 2 [file 44319_2025_443_MOESM5_ESM.zip › Figure 2/2F/CHX/+HU.tif]

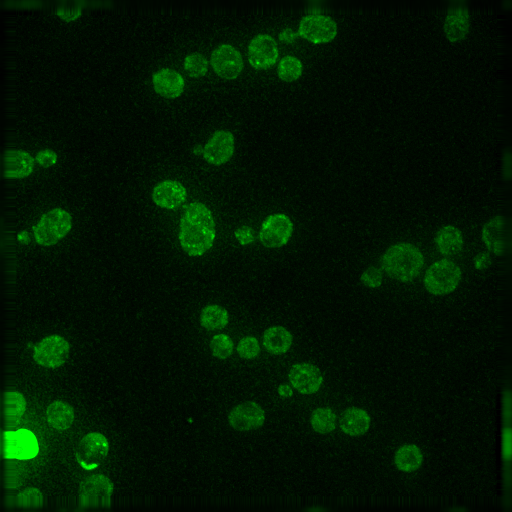

Supplement: Supplementary file 5 — Source data Fig. 2 [file 44319_2025_443_MOESM5_ESM.zip › Figure 2/2F/CHX/-HU.tif]

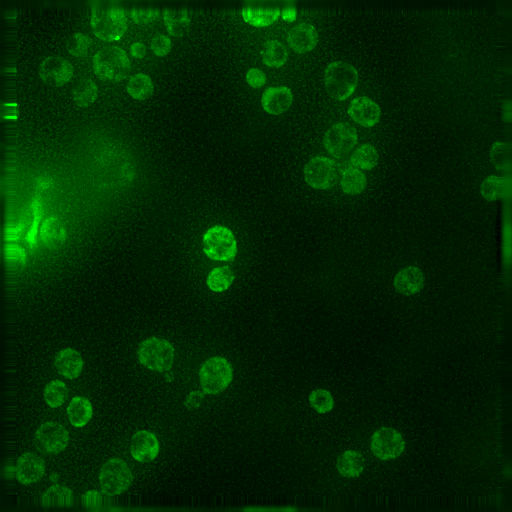

Supplement: Supplementary file 5 — Source data Fig. 2 [file 44319_2025_443_MOESM5_ESM.zip › Figure 2/2F/CHX/HU+CHX.tif]

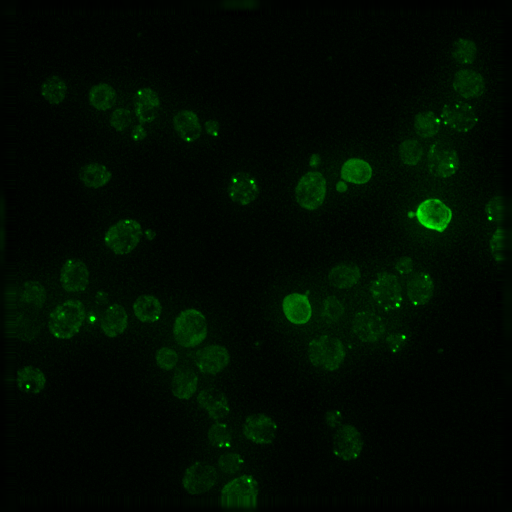

Supplement: Supplementary file 5 — Source data Fig. 2 [file 44319_2025_443_MOESM5_ESM.zip › Figure 2/2F/Recovery/+HU.tif]

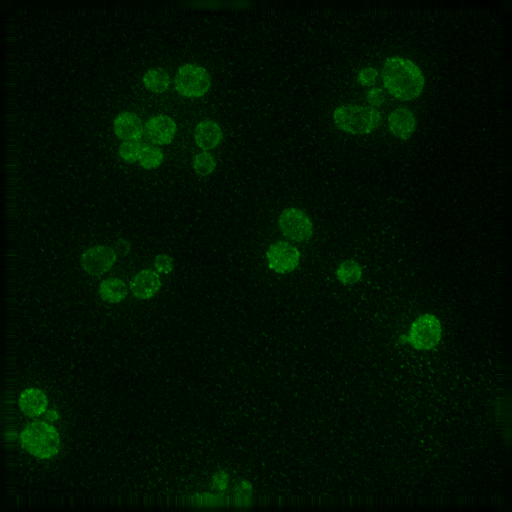

Supplement: Supplementary file 5 — Source data Fig. 2 [file 44319_2025_443_MOESM5_ESM.zip › Figure 2/2F/Recovery/-HU.tif]

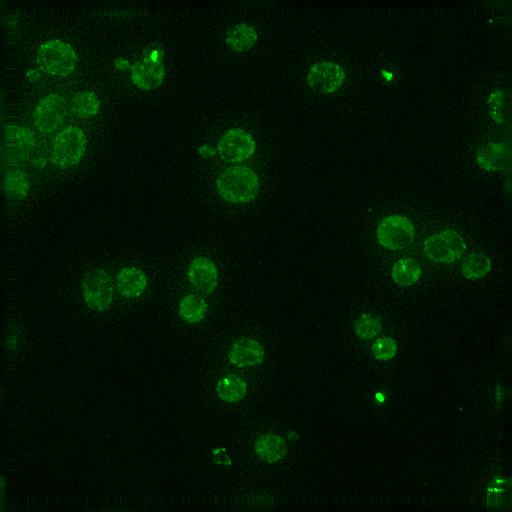

Supplement: Supplementary file 5 — Source data Fig. 2 [file 44319_2025_443_MOESM5_ESM.zip › Figure 2/2F/Recovery/Recovery.tif]

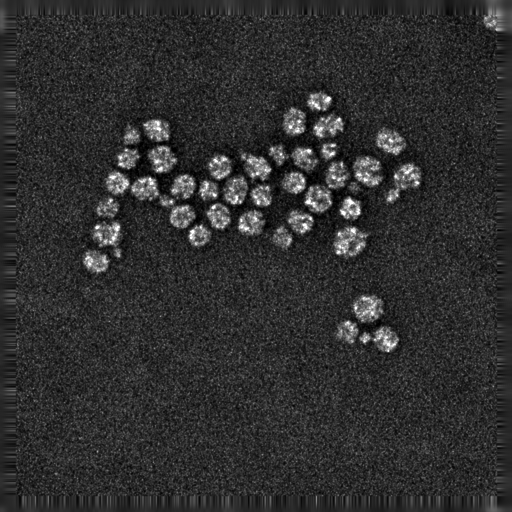

Supplement: Supplementary file 6 — Source data Fig. 3 [file 44319_2025_443_MOESM6_ESM.zip › Figure 3/3A/+HU/C2-SRS2 mRNA_Cy5.tif]

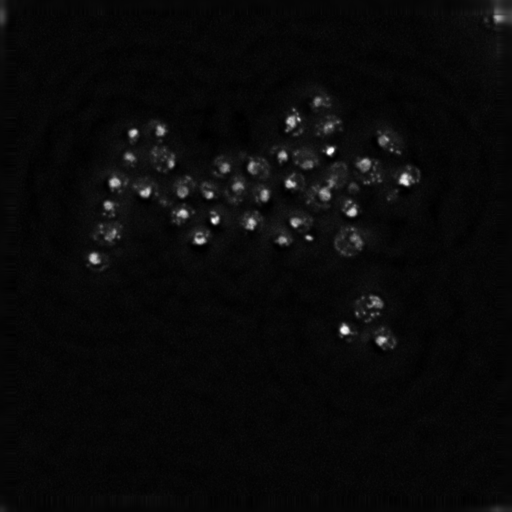

Supplement: Supplementary file 6 — Source data Fig. 3 [file 44319_2025_443_MOESM6_ESM.zip › Figure 3/3A/+HU/DAPI.tif]

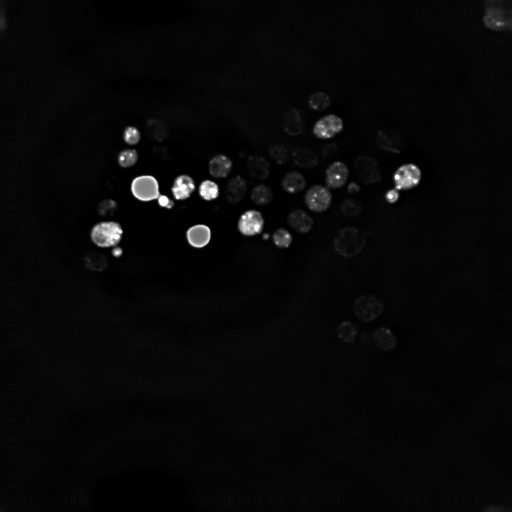

Supplement: Supplementary file 6 — Source data Fig. 3 [file 44319_2025_443_MOESM6_ESM.zip › Figure 3/3A/+HU/merge.tif]

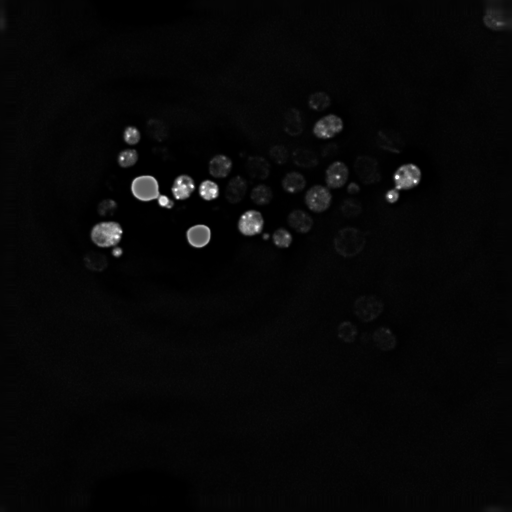

Supplement: Supplementary file 6 — Source data Fig. 3 [file 44319_2025_443_MOESM6_ESM.zip › Figure 3/3A/+HU/Scd6GFP.tif]

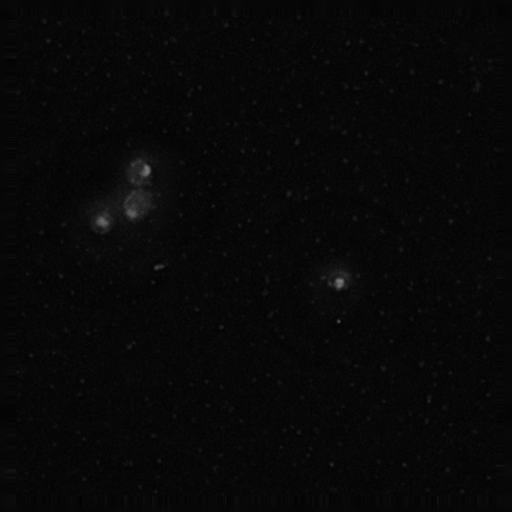

Supplement: Supplementary file 6 — Source data Fig. 3 [file 44319_2025_443_MOESM6_ESM.zip › Figure 3/3A/-HU/DAPI.tif]

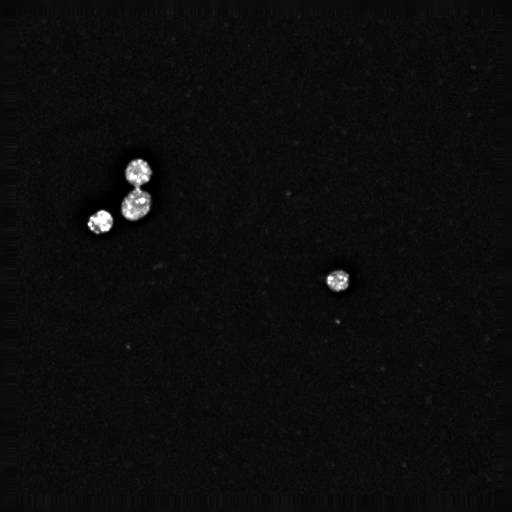

Supplement: Supplementary file 6 — Source data Fig. 3 [file 44319_2025_443_MOESM6_ESM.zip › Figure 3/3A/-HU/Merge.tif]

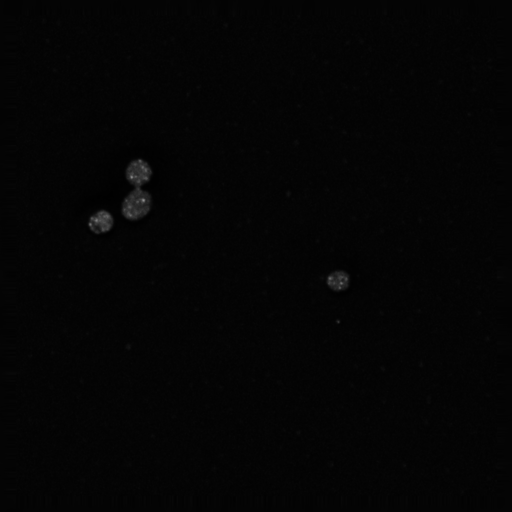

Supplement: Supplementary file 6 — Source data Fig. 3 [file 44319_2025_443_MOESM6_ESM.zip › Figure 3/3A/-HU/Scd6GFP.tif]

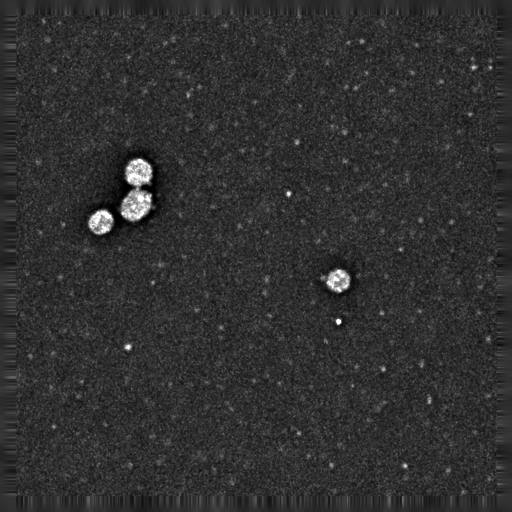

Supplement: Supplementary file 6 — Source data Fig. 3 [file 44319_2025_443_MOESM6_ESM.zip › Figure 3/3A/-HU/SRS2 mRNA_cy5.tif]

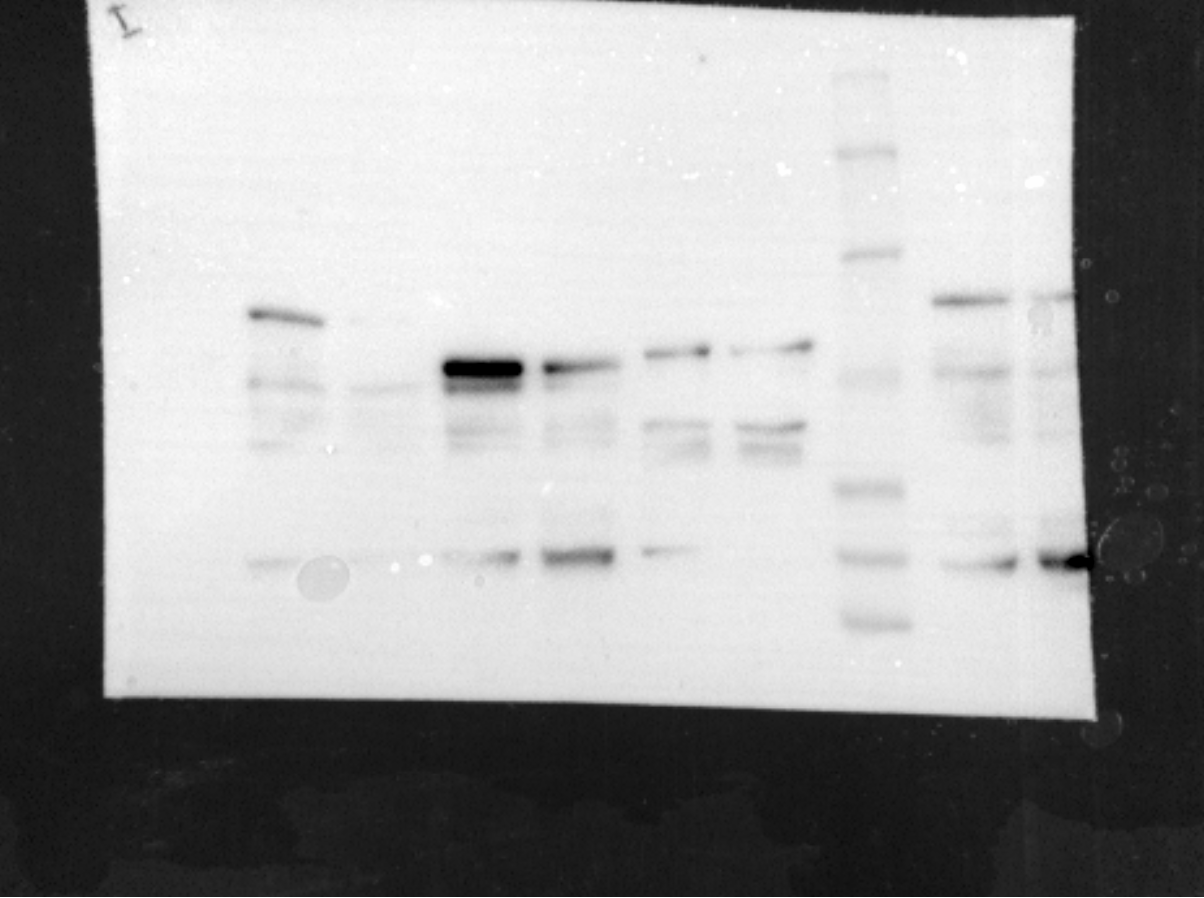

Supplement: Supplementary file 7 — Source data Fig. 4 [file 44319_2025_443_MOESM7_ESM.zip › Figure 4/4B/GFP Input.tif]

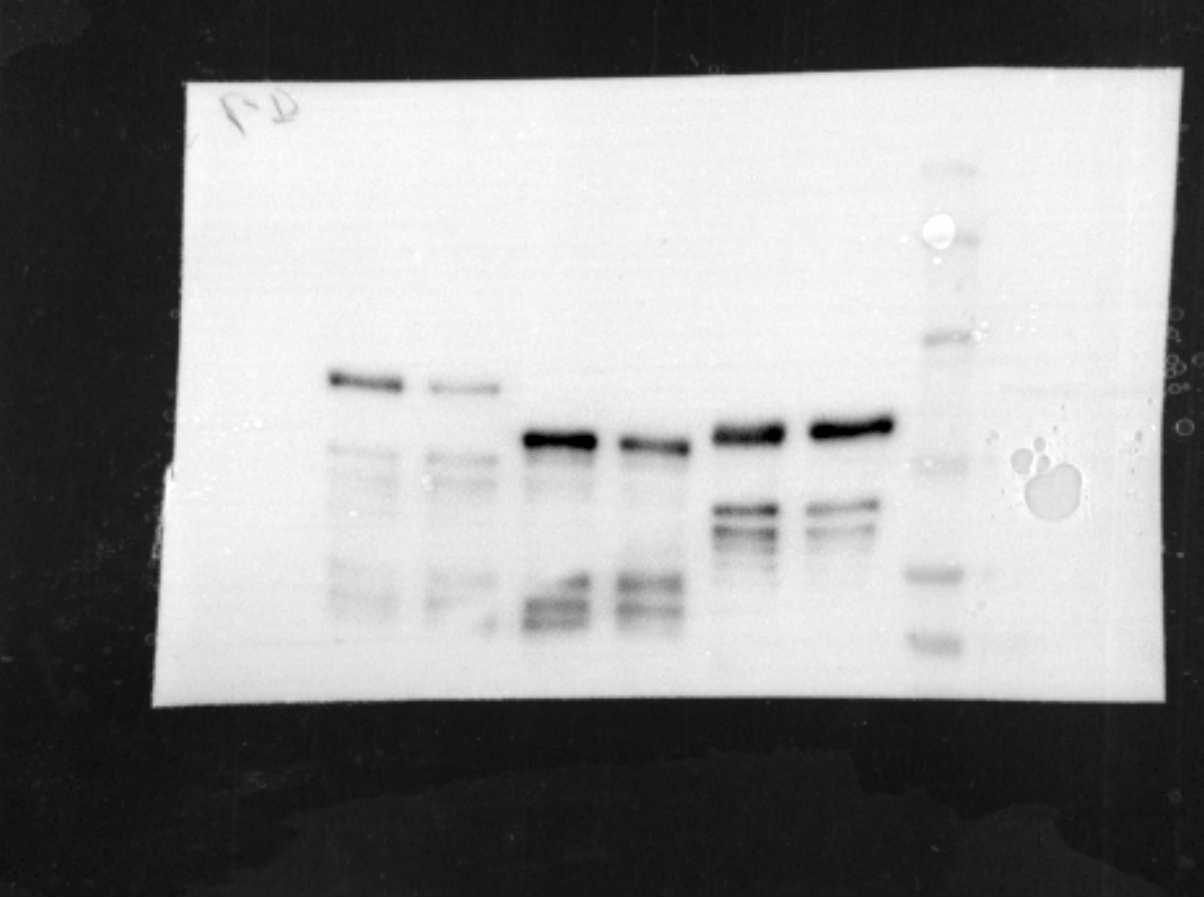

Supplement: Supplementary file 7 — Source data Fig. 4 [file 44319_2025_443_MOESM7_ESM.zip › Figure 4/4B/GFP PD.tif]

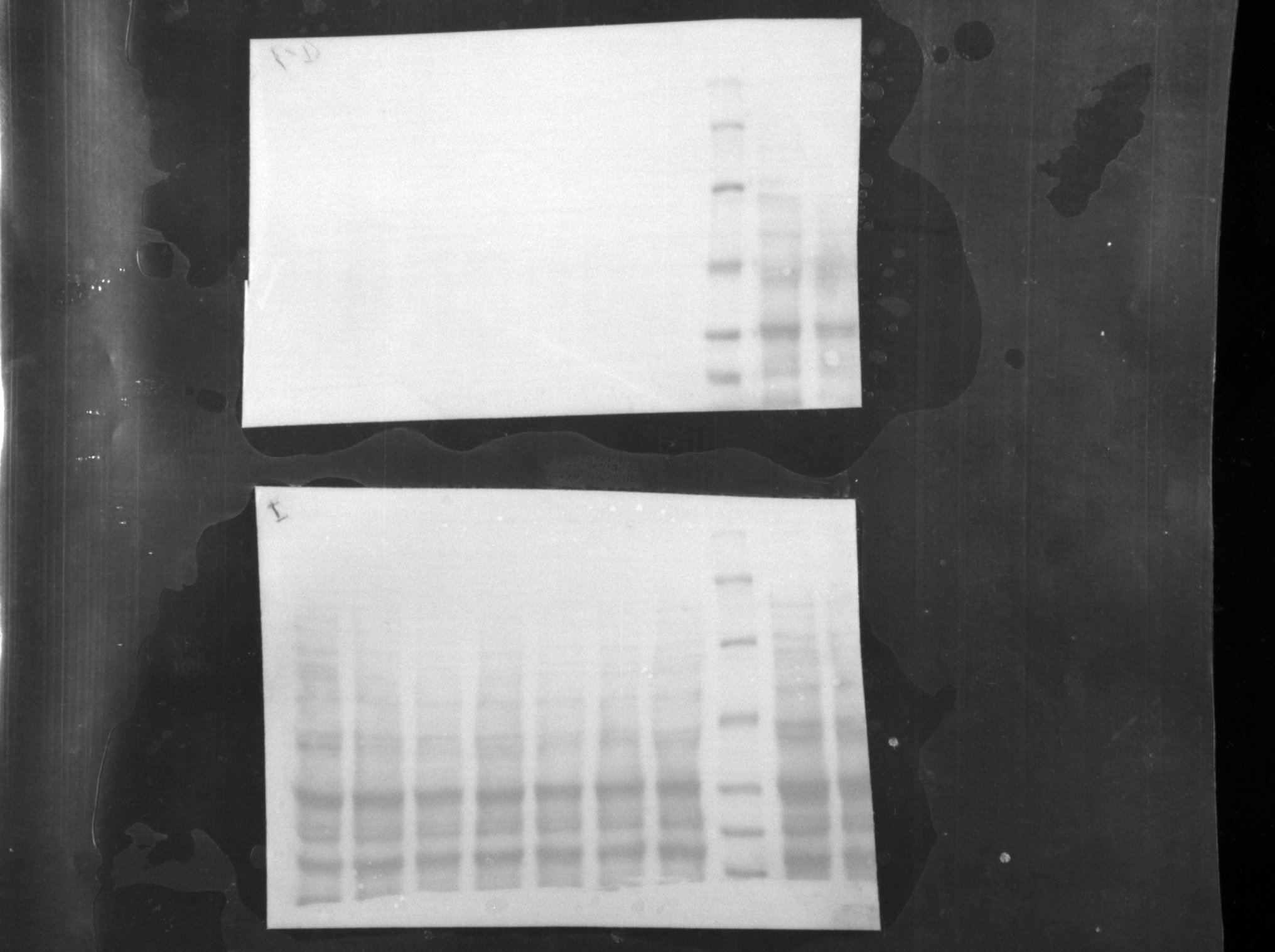

Supplement: Supplementary file 7 — Source data Fig. 4 [file 44319_2025_443_MOESM7_ESM.zip › Figure 4/4B/ponceau.tif]

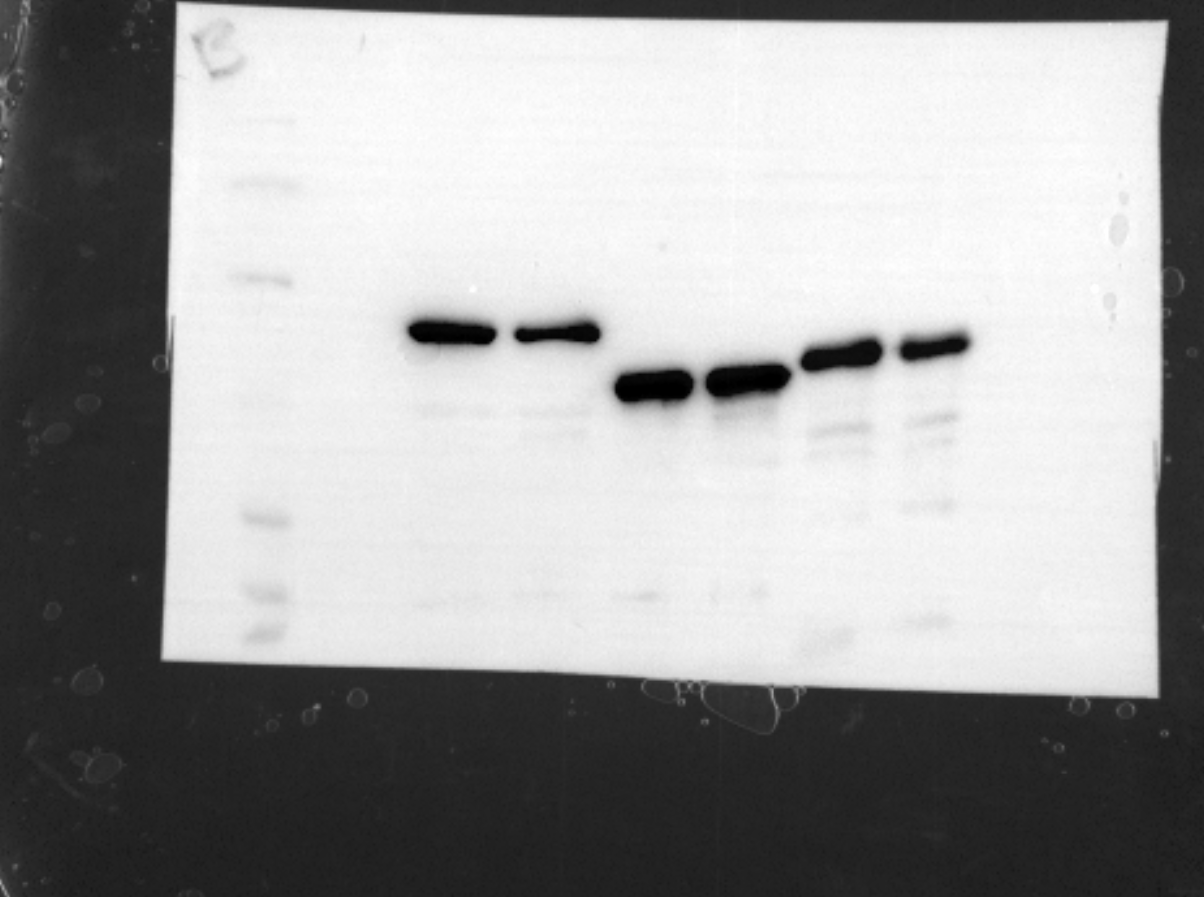

Supplement: Supplementary file 7 — Source data Fig. 4 [file 44319_2025_443_MOESM7_ESM.zip › Figure 4/4D/anti-GFP.tif]

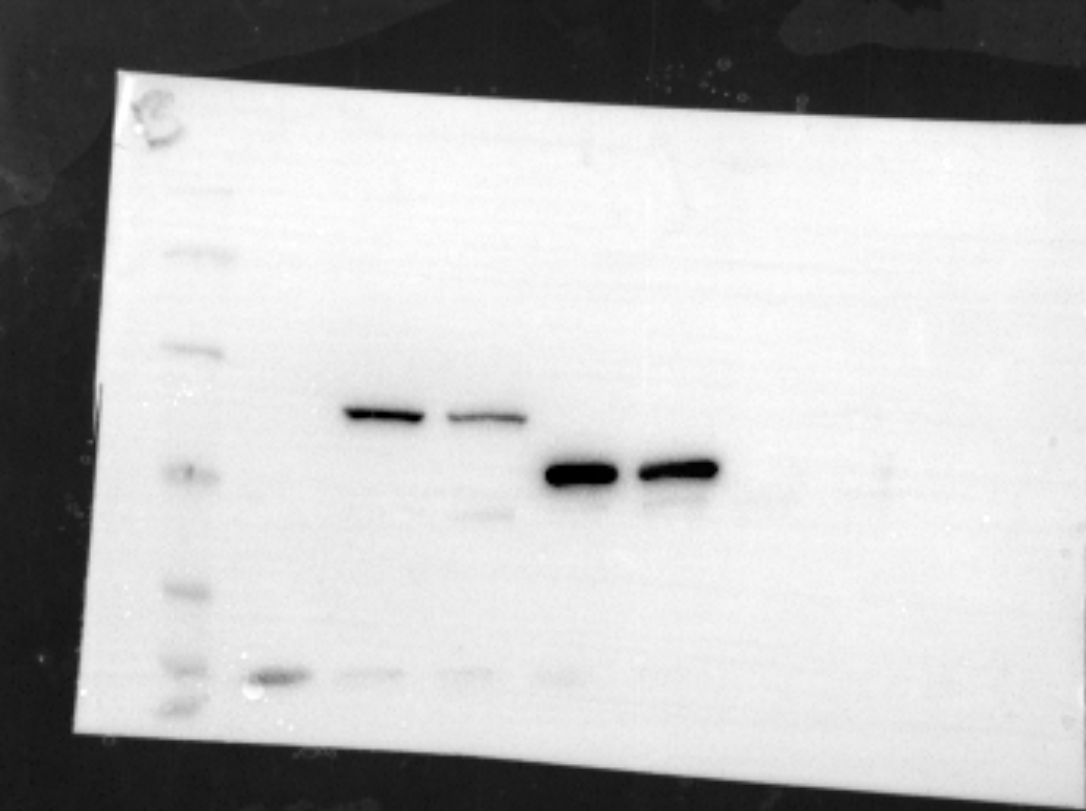

Supplement: Supplementary file 7 — Source data Fig. 4 [file 44319_2025_443_MOESM7_ESM.zip › Figure 4/4D/anti-MMA.tif]

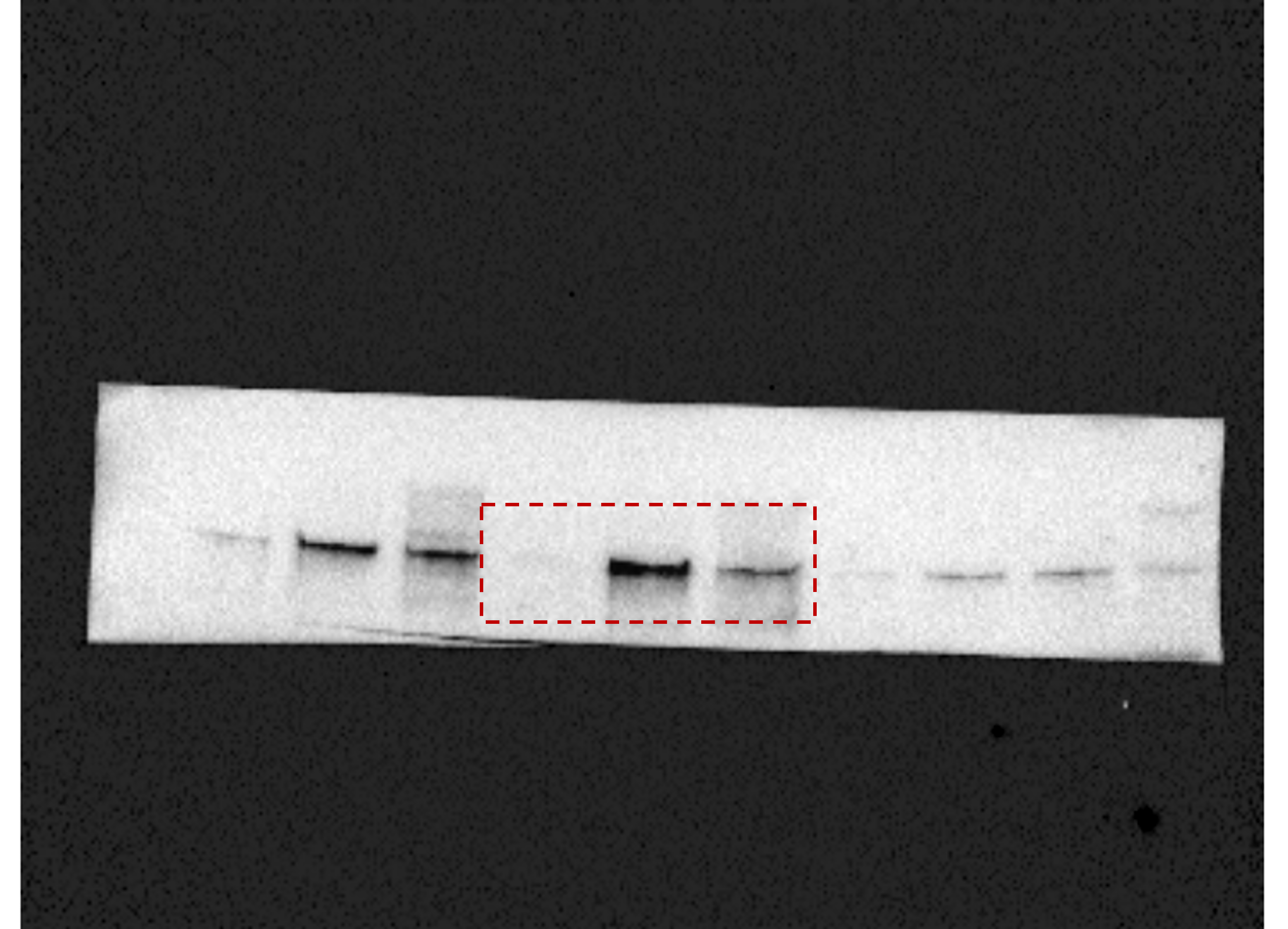

Supplement: Supplementary file 7 — Source data Fig. 4 [file 44319_2025_443_MOESM7_ESM.zip › Figure 4/4F/4F_anti eIF4G1.tif]

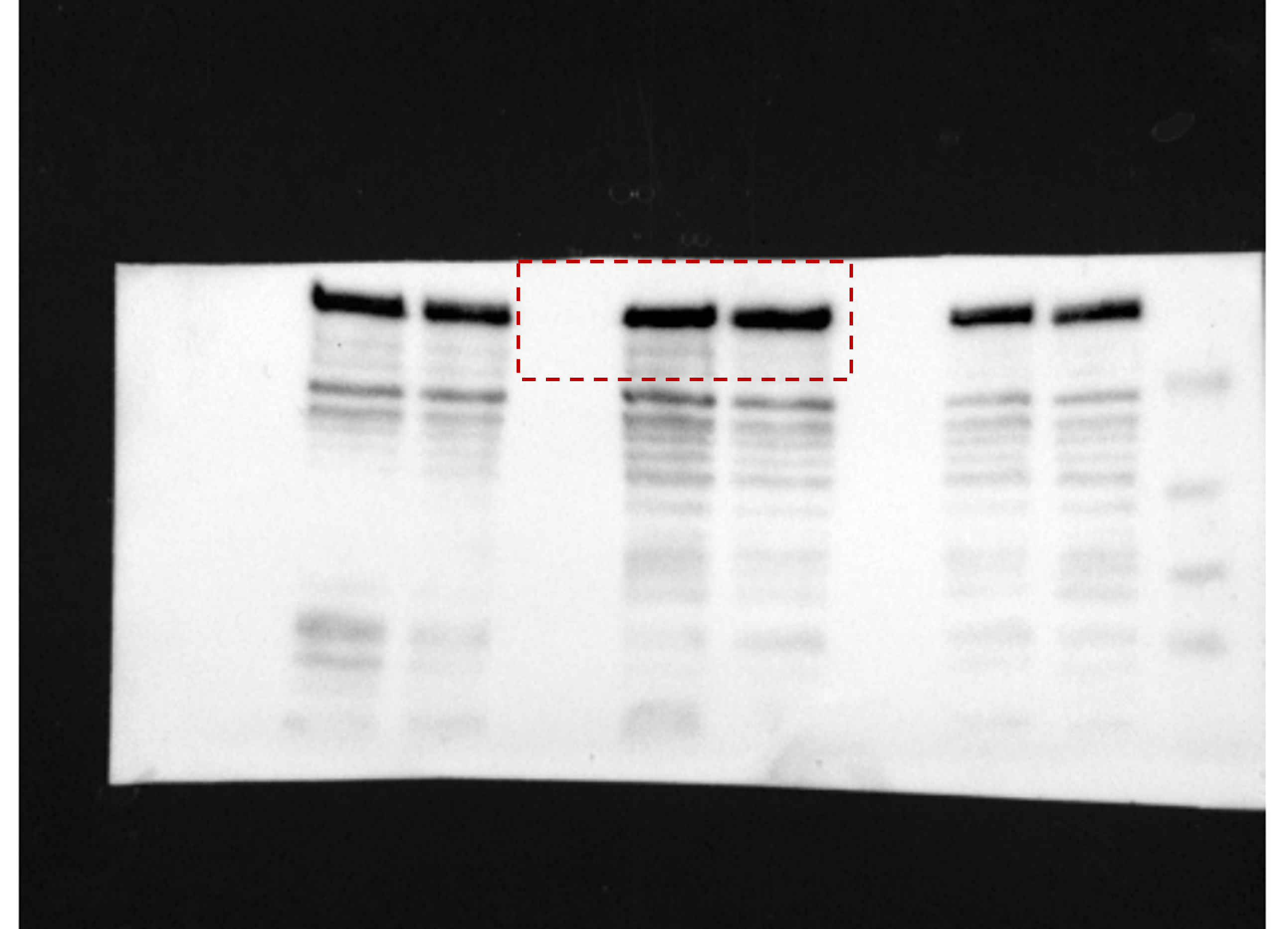

Supplement: Supplementary file 7 — Source data Fig. 4 [file 44319_2025_443_MOESM7_ESM.zip › Figure 4/4F/4F_anti GST.tif]

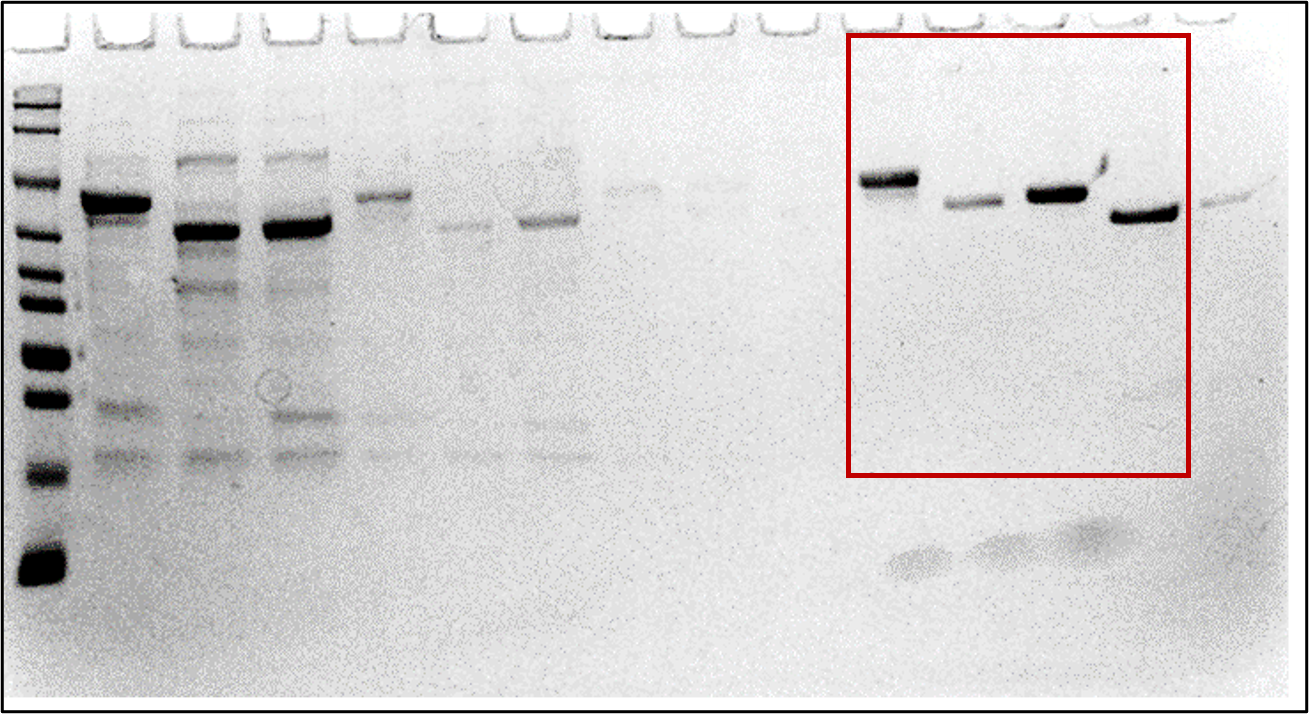

Supplement: Supplementary file 7 — Source data Fig. 4 [file 44319_2025_443_MOESM7_ESM.zip › Figure 4/4H/4H_CBB gel.tif]

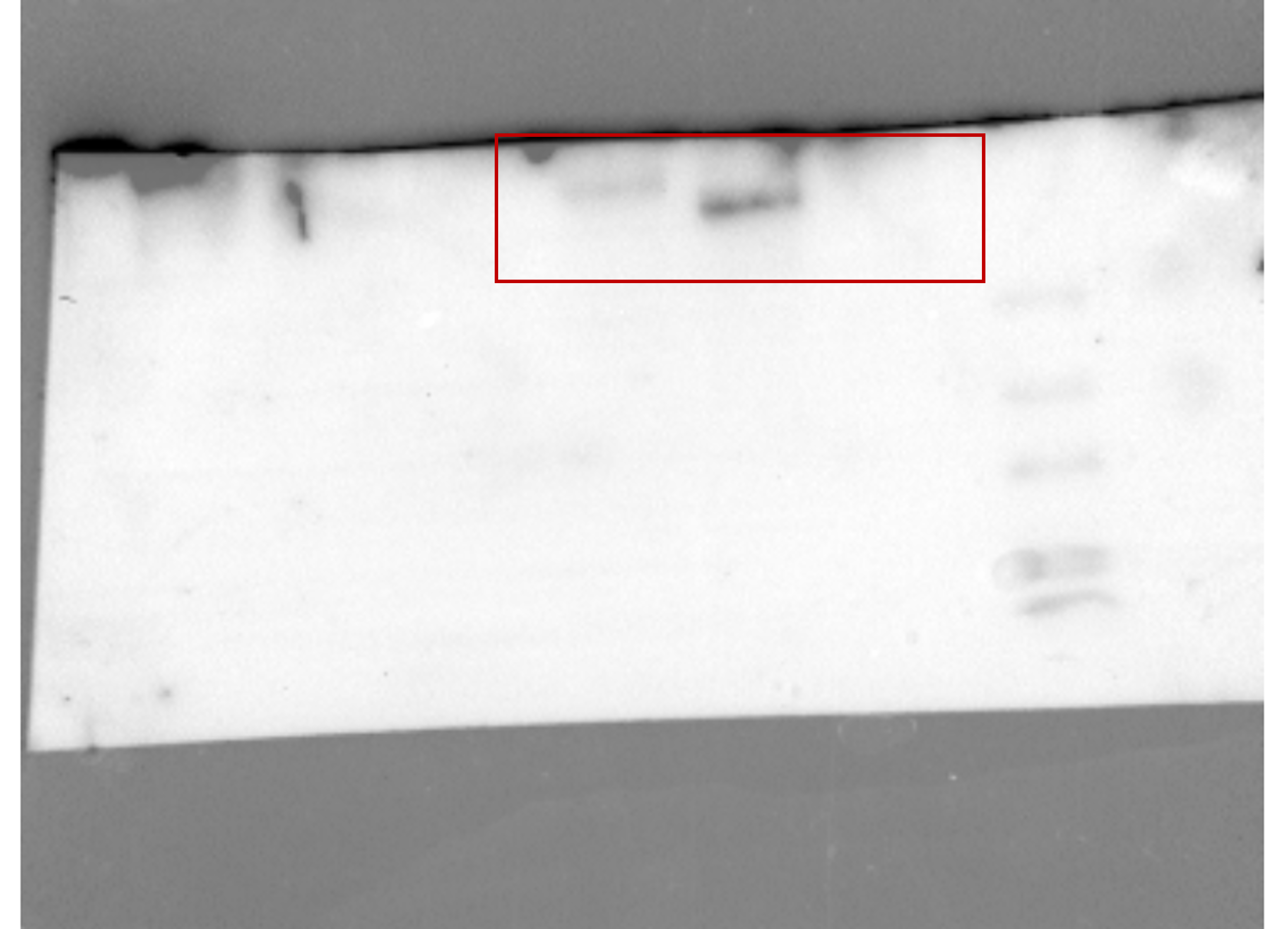

Supplement: Supplementary file 7 — Source data Fig. 4 [file 44319_2025_443_MOESM7_ESM.zip › Figure 4/4I/4I_anti MMA.tif]

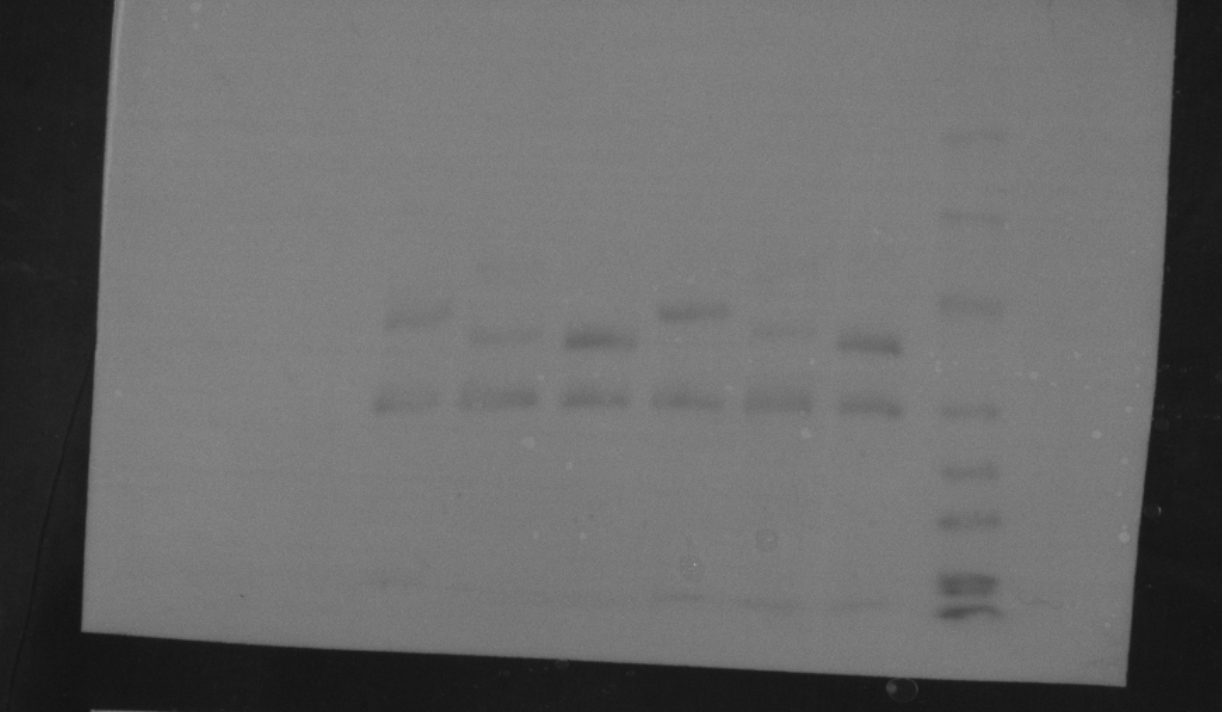

Supplement: Supplementary file 7 — Source data Fig. 4 [file 44319_2025_443_MOESM7_ESM.zip › Figure 4/4I/4I_Ponceau.tif]

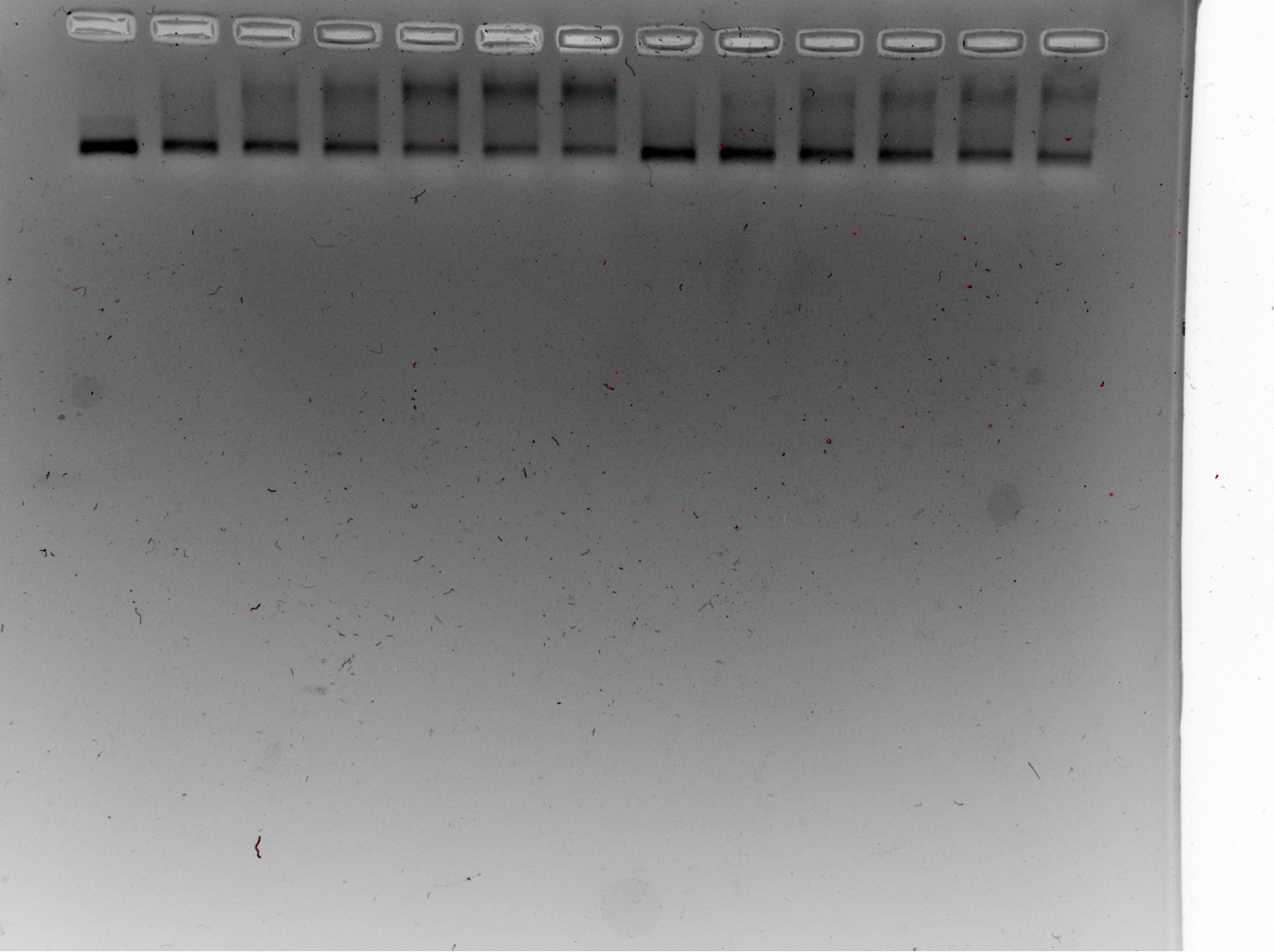

Supplement: Supplementary file 7 — Source data Fig. 4 [file 44319_2025_443_MOESM7_ESM.zip › Figure 4/4K/4K_gel image.tif]

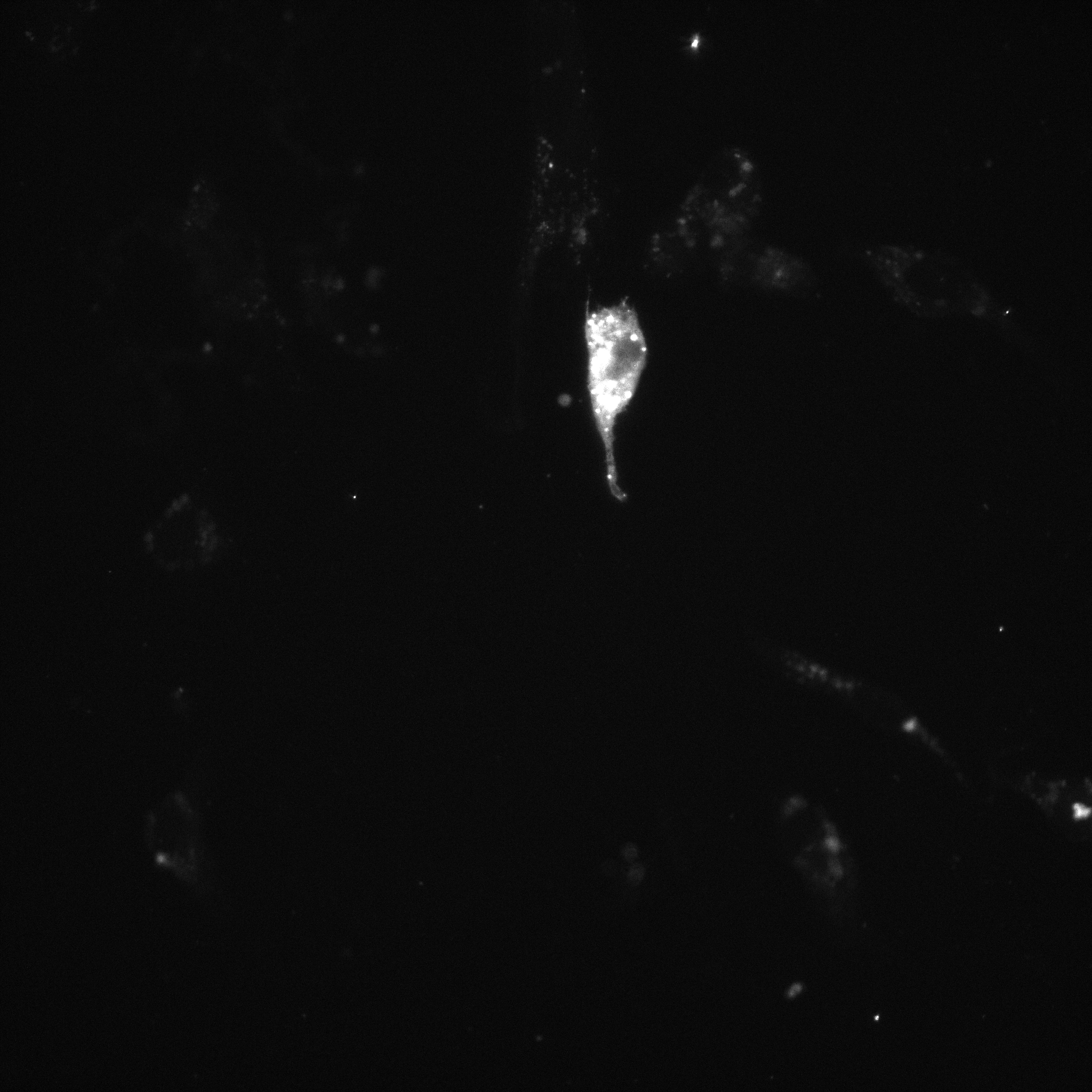

Supplement: Supplementary file 8 — Source data Fig. 5 [file 44319_2025_443_MOESM8_ESM.zip › Figure 5/5B/delRGG+HU/IF_LSM14A-GFP_Delta R1+2_HU_1_w11 FITC.TIF]

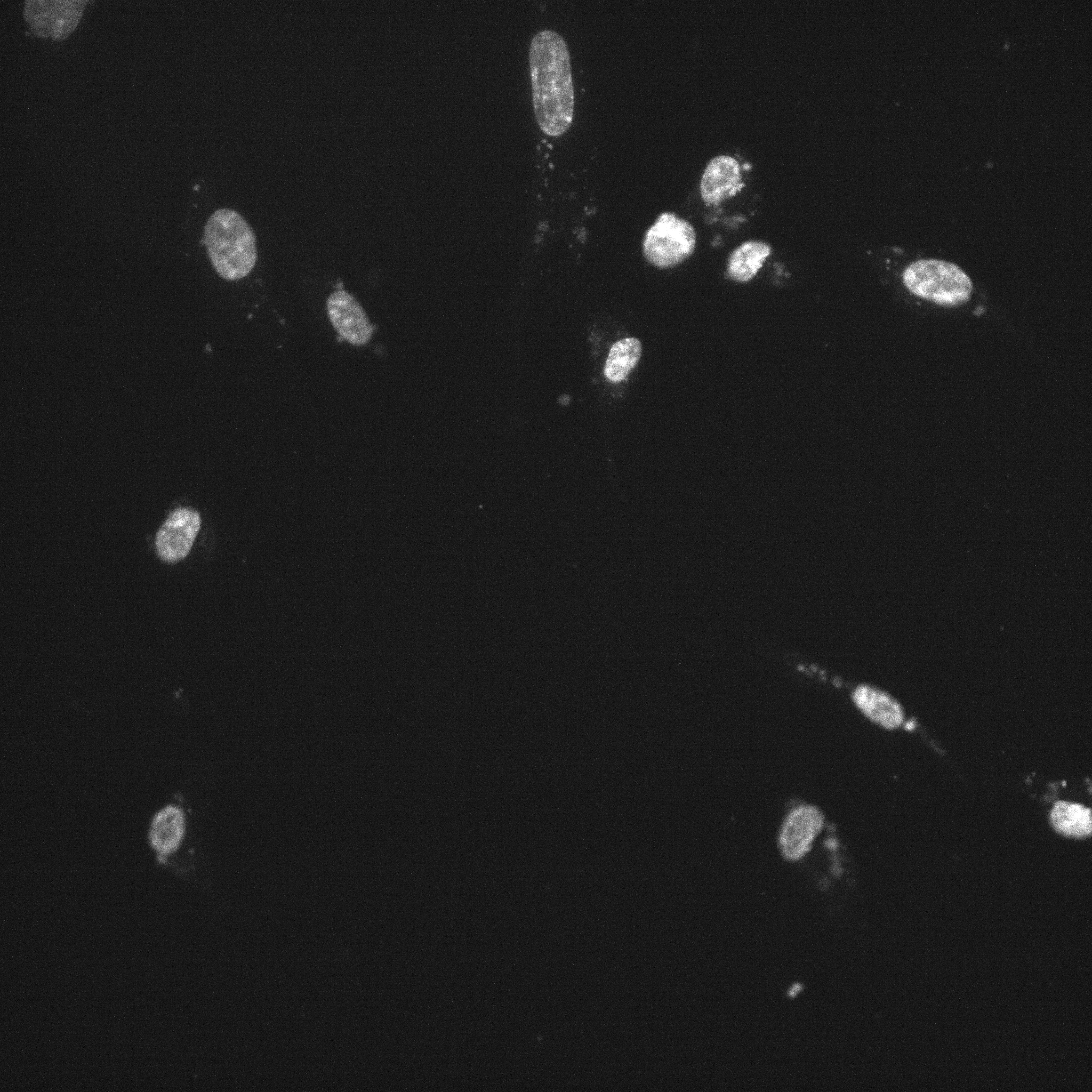

Supplement: Supplementary file 8 — Source data Fig. 5 [file 44319_2025_443_MOESM8_ESM.zip › Figure 5/5B/delRGG+HU/IF_LSM14A-GFP_Delta R1+2_HU_1_w21 DAPI 405.TIF]

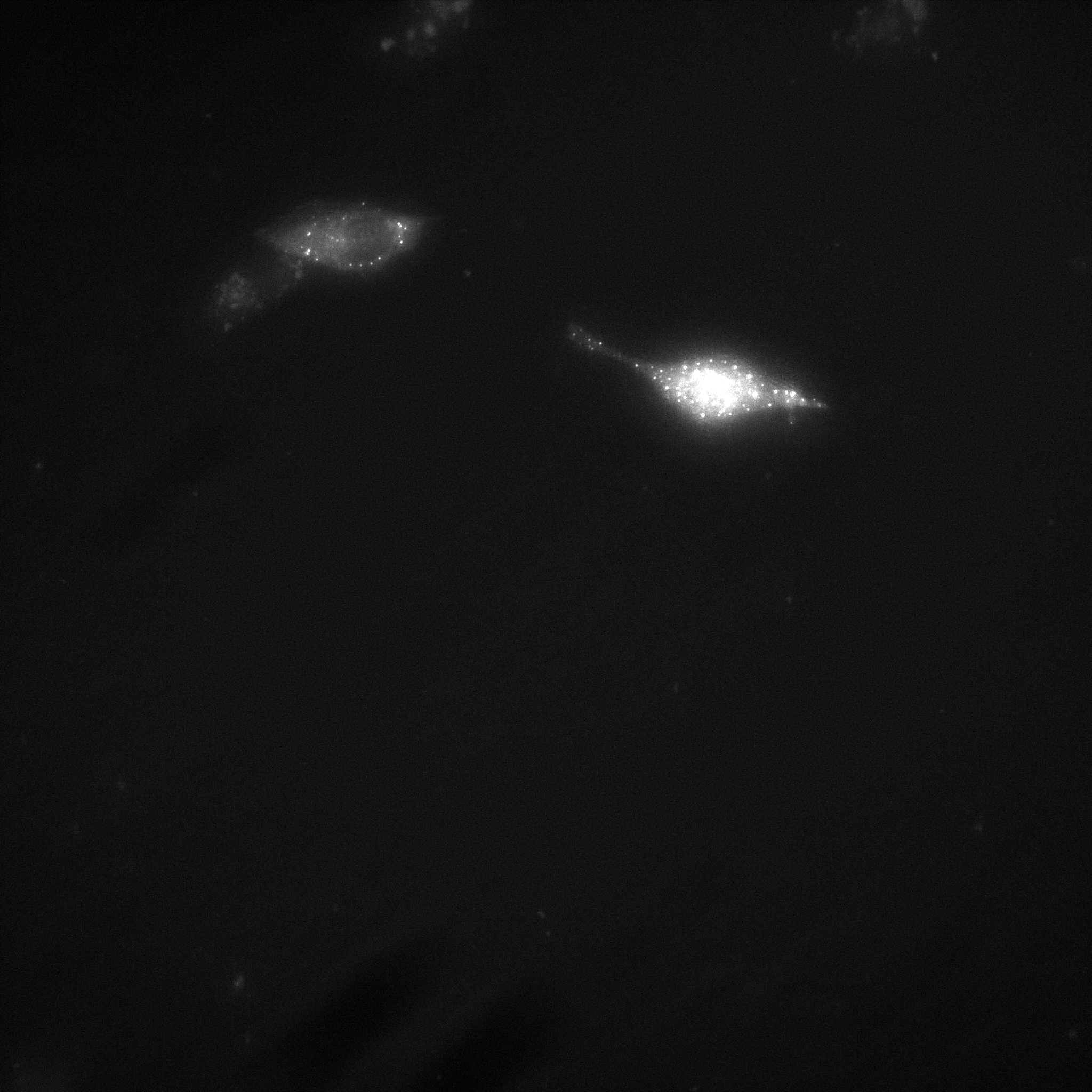

Supplement: Supplementary file 8 — Source data Fig. 5 [file 44319_2025_443_MOESM8_ESM.zip › Figure 5/5B/delRGG-HU/IF_LSM14A-GFP_DeltaR1+2_UT_1_w11 FITC.TIF]

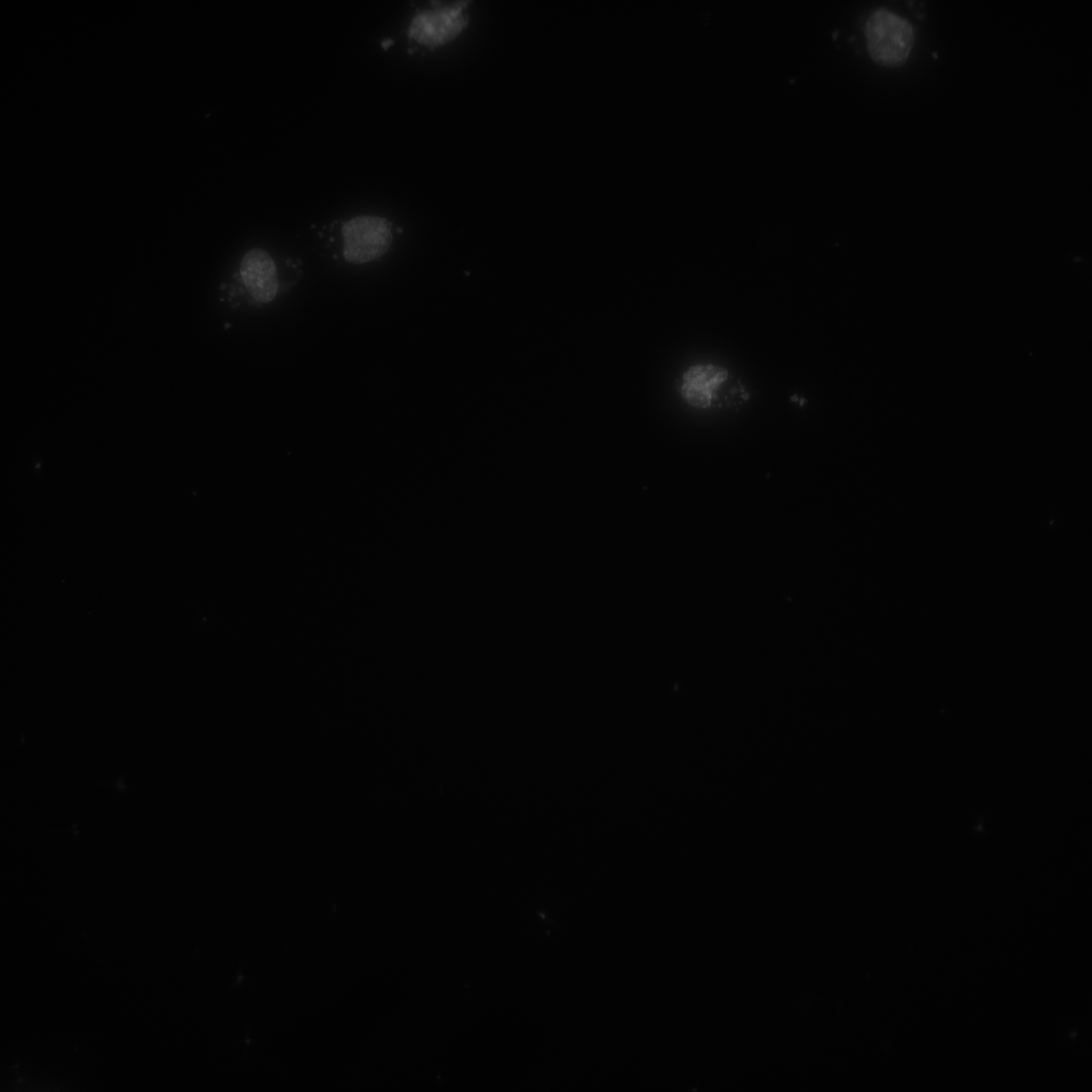

Supplement: Supplementary file 8 — Source data Fig. 5 [file 44319_2025_443_MOESM8_ESM.zip › Figure 5/5B/delRGG-HU/IF_LSM14A-GFP_DeltaR1+2_UT_1_w21 DAPI 405.TIF]

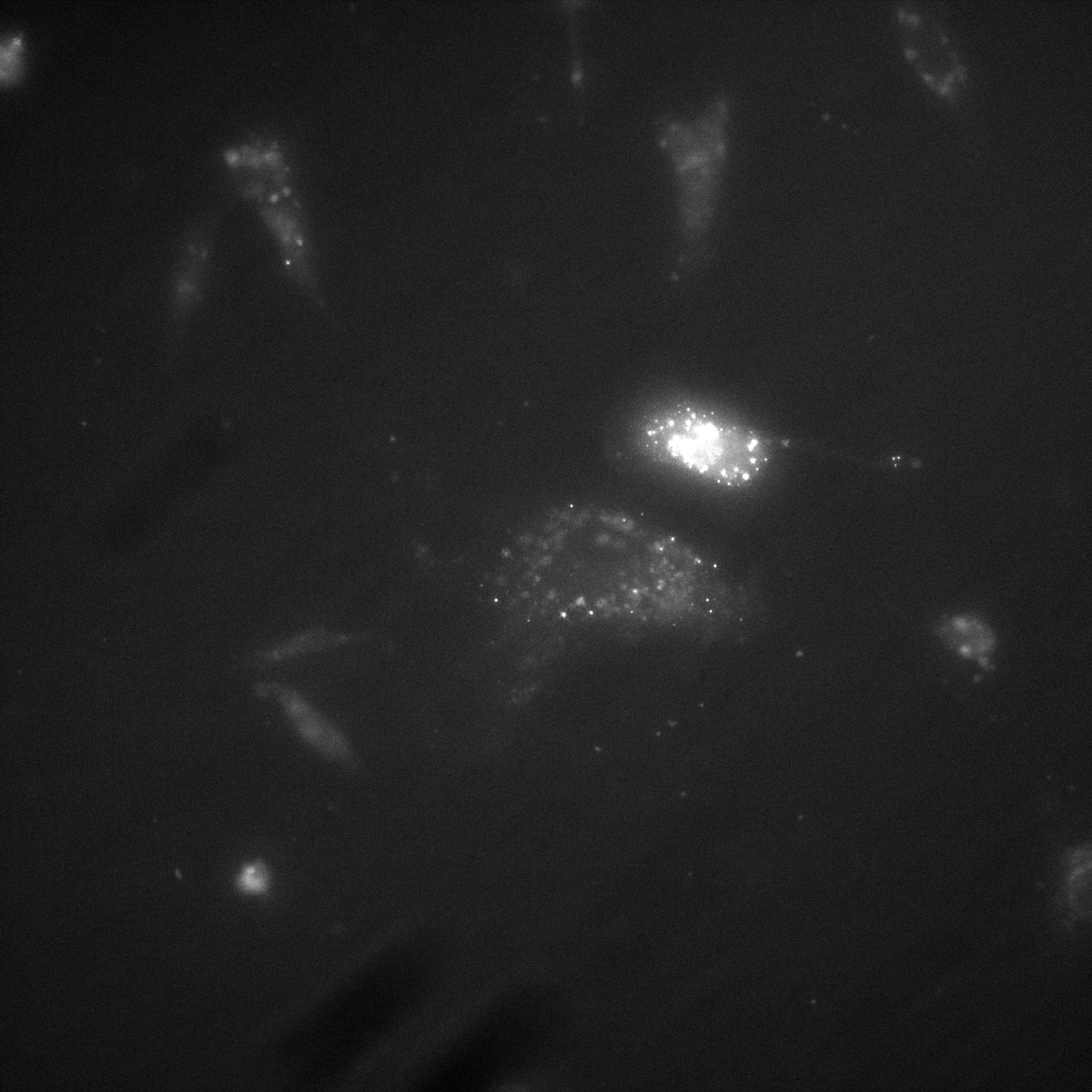

Supplement: Supplementary file 8 — Source data Fig. 5 [file 44319_2025_443_MOESM8_ESM.zip › Figure 5/5B/WT+HU/IF_LSM14A-GFP_WT_HU treated_11_w11 FITC.TIF]

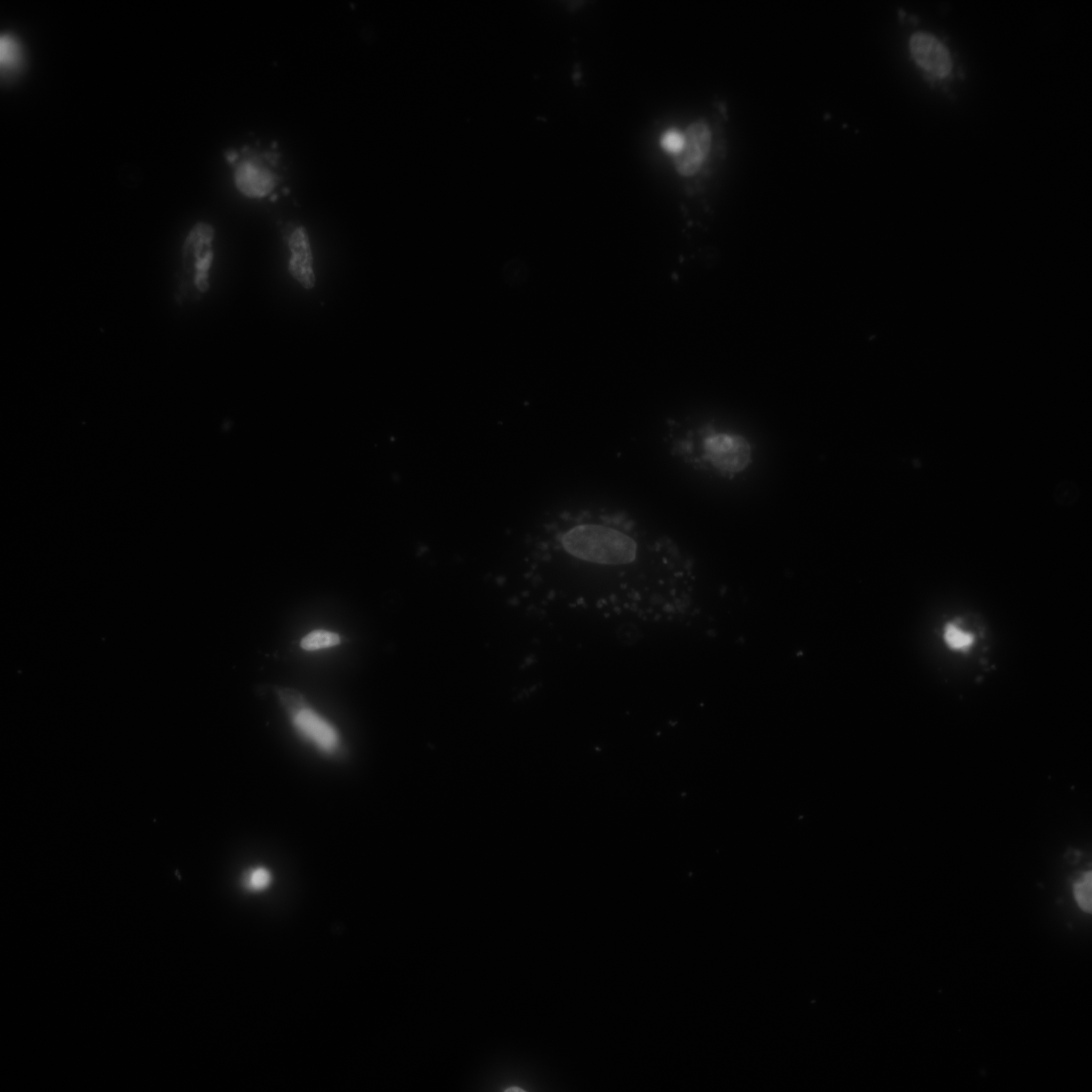

Supplement: Supplementary file 8 — Source data Fig. 5 [file 44319_2025_443_MOESM8_ESM.zip › Figure 5/5B/WT+HU/IF_LSM14A-GFP_WT_HU treated_11_w21 DAPI 405.TIF]

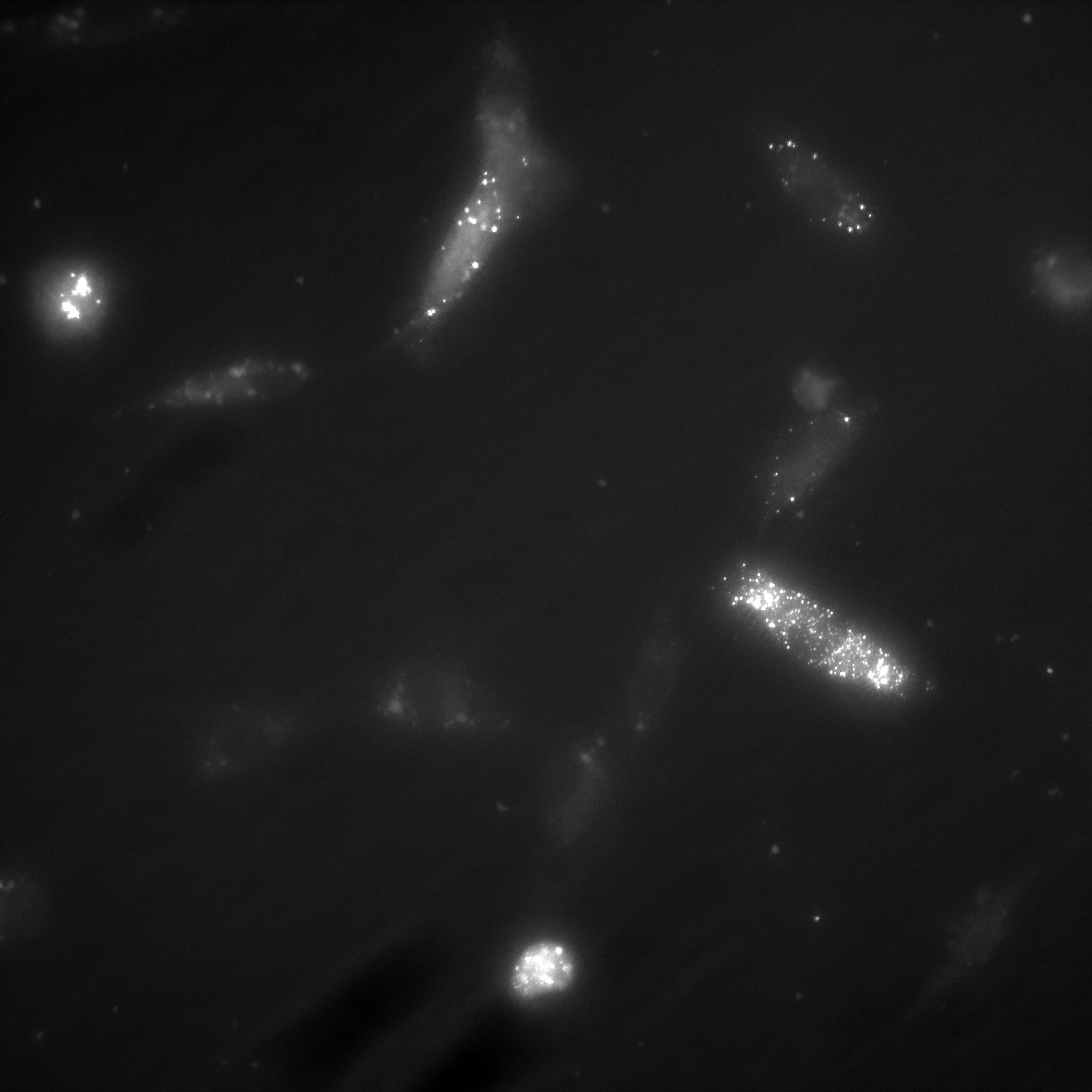

Supplement: Supplementary file 8 — Source data Fig. 5 [file 44319_2025_443_MOESM8_ESM.zip › Figure 5/5B/WT-HU/IF_LSM14A-GFP_WT_UT1_w11 FITC.TIF]

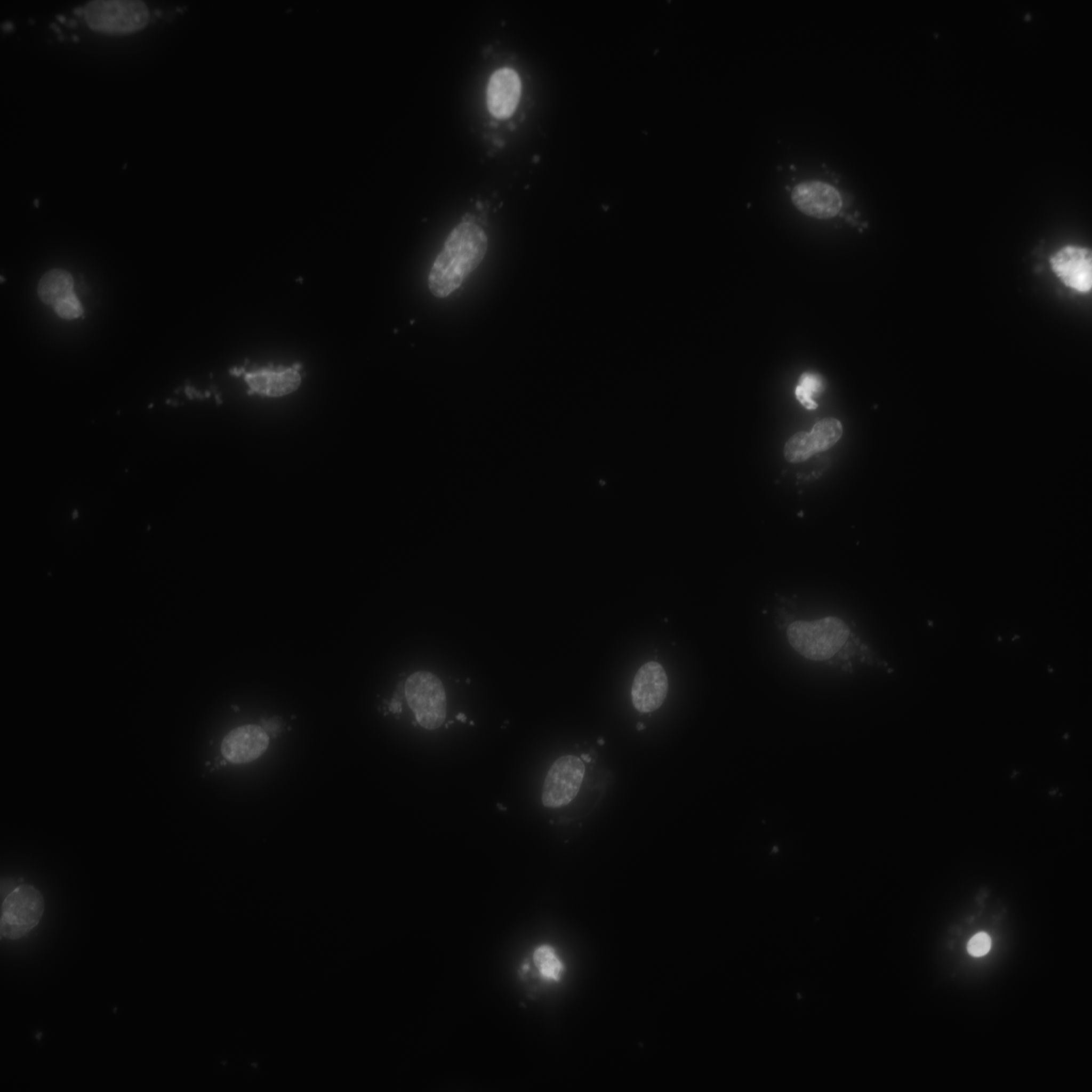

Supplement: Supplementary file 8 — Source data Fig. 5 [file 44319_2025_443_MOESM8_ESM.zip › Figure 5/5B/WT-HU/IF_LSM14A-GFP_WT_UT1_w21 DAPI 405.TIF]

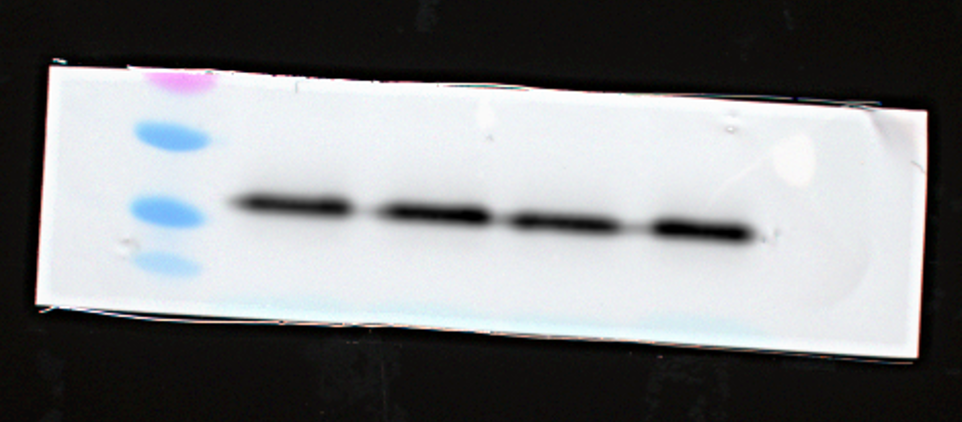

Supplement: Supplementary file 8 — Source data Fig. 5 [file 44319_2025_443_MOESM8_ESM.zip › Figure 5/5G/Histone3.tif]

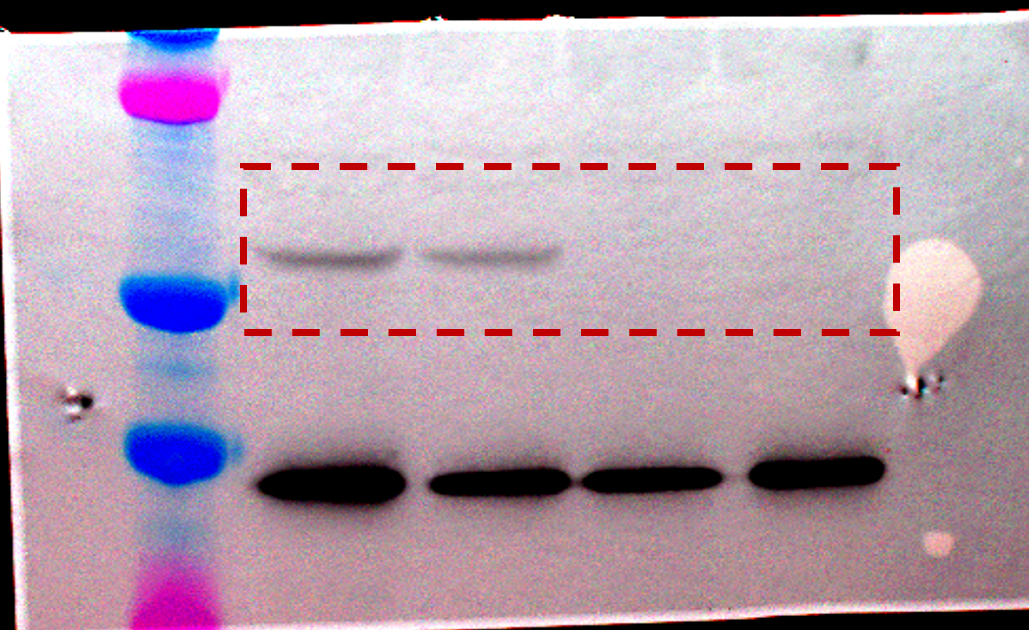

Supplement: Supplementary file 8 — Source data Fig. 5 [file 44319_2025_443_MOESM8_ESM.zip › Figure 5/5G/LSM14A.png]

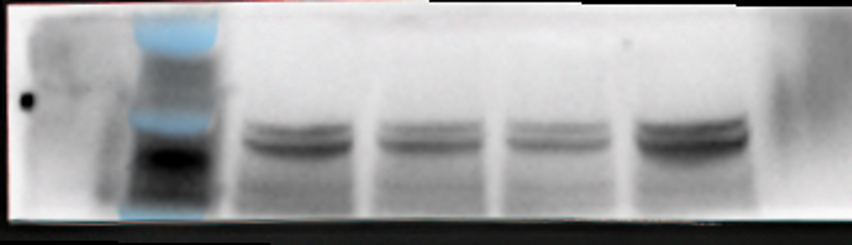

Supplement: Supplementary file 8 — Source data Fig. 5 [file 44319_2025_443_MOESM8_ESM.zip › Figure 5/5G/RTEL1.tif]
