# Supplementary material for: Genotoxic stress triggers Scd6-dependent regulation of translation to modulate the DNA damage response
Source: EMBO Rep. 2025 Apr 24;26(10):2715–39. doi: 10.1038/s44319-025-00443-3 (PMC12116771; doi:10.1038/s44319-025-00443-3)
Supplement: Supplementary file 9 — Expanded View Figures [file 44319_2025_443_MOESM9_ESM.pdf]

Expanded View Figures

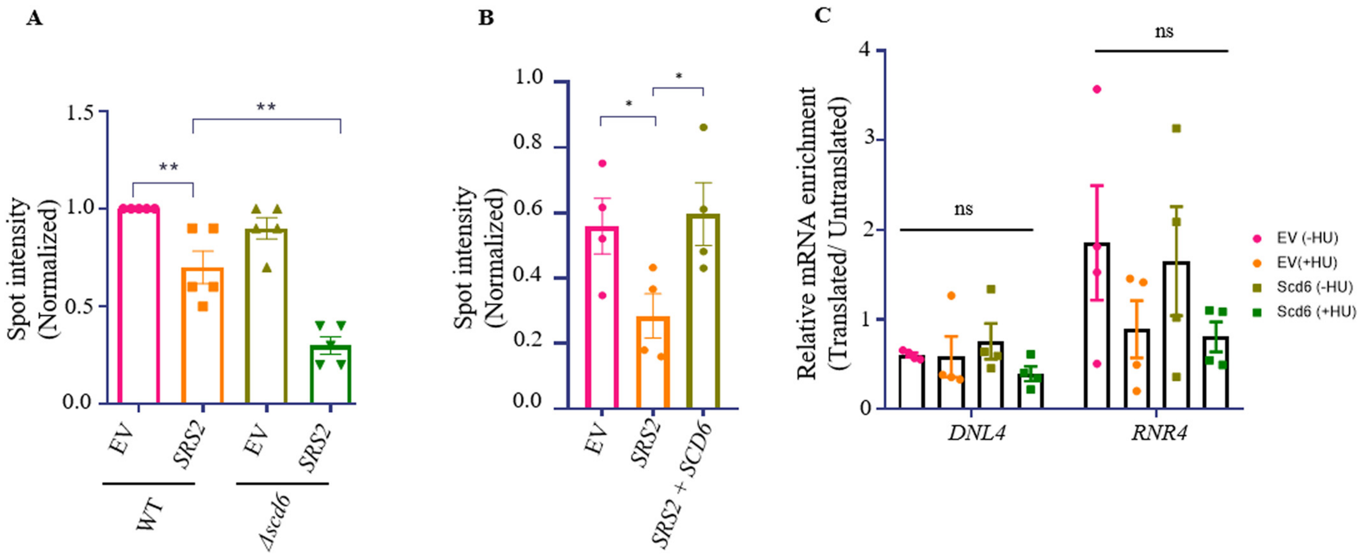

Figure EV1. Quantitiation of the SRS2-SCD6 genetic interaction.

(**A**) Quantification of growth assay (in Fig. 2A) by measuring the intensity of the second spot and normalizing the values of spots on HU plate to the respective control values for each strain ($n = 5$ biological replicates). Error bars indicate standard error of mean and statistical significance was calculated using unpaired t-test. WT EV vs WT SRS2, **$p = 0.0071$; WT SRS2 vs *Δscd6* SRS2, **$p = 0.0029$. (**B**) Quantification of growth assay (in Fig. 2C) by measuring the intensity of the third spot and normalizing the values of spots on HU plate to the respective control values for each strain ($n = 4$ biological replicates). Error bars indicate standard error of mean and statistical significance was calculated using unpaired t-test. EV vs SRS2, *$p = 0.0448$; SRS2 vs SRS2+Scd6, *$p = 0.0382$. (**C**) Quantification of *DNL4* and *RNR4* mRNA in the polysome fractions plotted as relative $\log_2$-Fold change ratio of Translated/Untranslated fractions ($n = 4$ biological replicates) using gene-specific primers. Error bars indicate standard error of mean and statistical significance was calculated using unpaired t-test.

 © The Author(s)

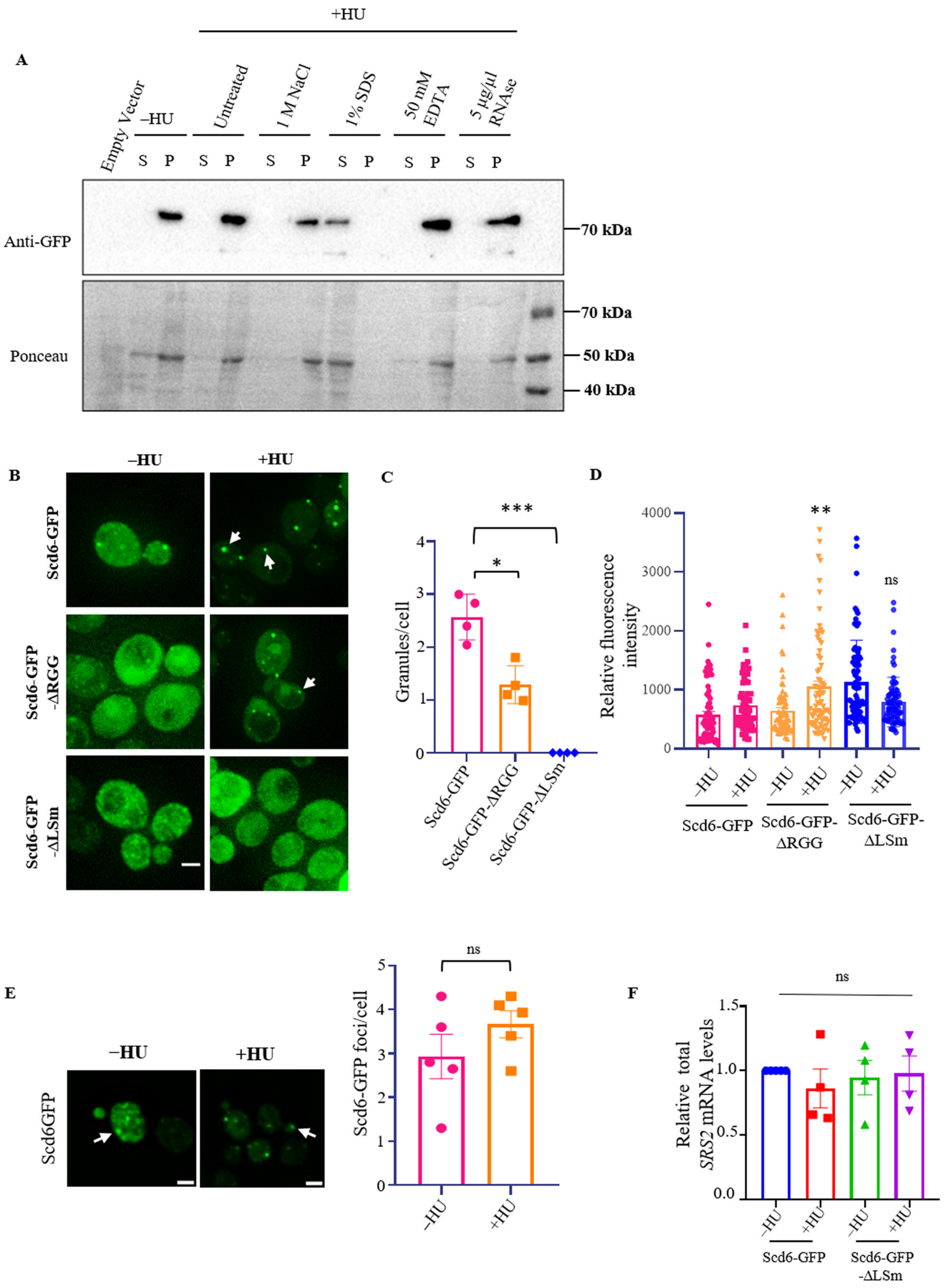
+HU
A
Empty Vector
–HU
Untreated
1 M NaCl
1% SDS
50 mM EDTA
5 μg/μl RNAse
S P S P S P S P S P S P
Anti-GFP
70 kDa
Ponceau
70 kDa
50 kDa
40 kDa
B
–HU
+HU
Scd6-GFP
Scd6-GFP -ΔRGG
Scd6-GFP -ΔLSm
C
***
*
Granules/cell
0
1
2
3
4
Scd6-GFP
Scd6-GFP-ΔRGG
Scd6-GFP-ΔLSm
D
**
ns
Relative fluorescence intensity
0
1000
2000
3000
4000
–HU
+HU
–HU
+HU
–HU
+HU
Scd6-GFP
Scd6-GFP-ΔRGG
Scd6-GFP-ΔLSm
E
–HU
+HU
Scd6GFP
ns
Scd6-GFP foci/cell
0
1
2
3
4
5
–HU
+HU
F
ns
Relative total SRS2 mRNA levels
0.0
0.5
1.0
1.5
–HU
+HU
–HU
+HU
Scd6-GFP
Scd6-GFP -ΔLSm

Figure EV2. Scd6 localizes to higher order mRNP condensates upon HU stress in LSm and RGG-motif dependent manner.

(**A**) Western blot showing partitioning of granule enriched Scd6GFP into soluble (S) or pellet (P) fraction upon treatment with 1M NaCl, 1% SDS, 50 mM EDTA or 5 µg/µl RNAse. (**B**) Live cell imaging showing localization of Scd6 and its domain deletion mutants upon HU treatment. White arrows indicate cytoplasmic foci. Scale bar = 2 µm. (**C**) Quantification of number of granules per cell shown in (**B**) ($n = 4$ biological replicates). Statistical significance was calculated using an unpaired t-test. Error bars indicate the standard error of the mean. Scd6-GFP vs Scd6GFP-ΔRGG, *$p = 0.0039$; Scd6-GFP vs Scd6GFP-ΔLSm ***$p = 0.0001$. (**D**) Quantification of relative GFP intensity as a measure of Scd6-GFP and mutants protein expression ($n = 3$ biological replicates, ≥70 cells were counted). Statistical significance was calculated using Tukey's multiple comparisons test. Error bars indicate the standard error of the mean. Scd6GFP-ΔLSm (−HU) vs Scd6GFP-ΔLSm (+HU), **$p = 0.0037$. (**E**) Live cell imaging showing granular localization of Scd6-GFP expressed on a 2 µ plasmid (left panel) and quantification of granule per cell localization of Scd6GFP (right panel) ($n = 5$ biological replicates, ≥100 cells counted). White arrows indicate cytoplasmic foci. Scale bar = 2 µm. (**F**) Quantification of relative total *SRS2* mRNA levels from lysate (left panel), and relative enrichment of Scd6 protein and *SRS2* mRNA in soluble (supernatant) and the heavier (granule-enriched fraction) fraction (right panel) (related to Fig. 3F) $n = 4$ biological replicates. Statistical significance was calculated using an unpaired t-test. Error bars indicate standard error of mean.

 © The Author(s)

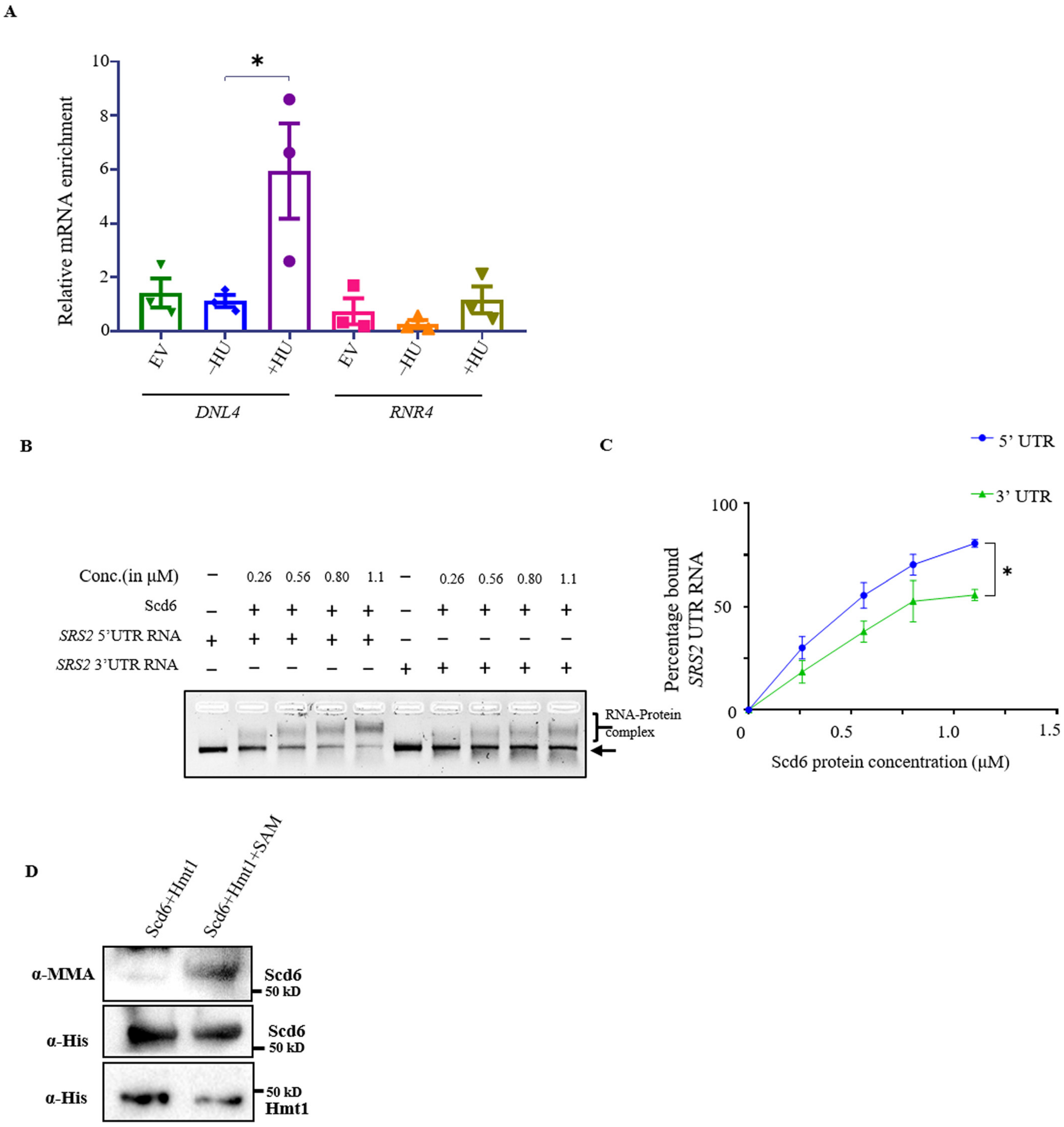

Figure EV3. Scd6 binds to specific mRNAs.

(**A**) Quantification of mRNA enrichment in RNA immunoprecipitation with *DNL4* and *RNR4* specific primers ($n = 3$ biological replicates). Statistical significance was calculated using unpaired t-test. Error bars indicate standard error of mean. $^*p = 0.0351$. (**B**) Ethidium Bromide (EtBr) stained agarose gel for electromobility shift assay (EMSA) with increasing concentrations of recombinant Scd6 incubated with 200-mer 5′UTR or 3′ UTR fragment of *SRS2 mRNA* (1.7 µM RNA) and its quantification in (**C**) ($n = 3$ technical replicates) Black arrow denotes unbound RNA. Statistical significance was calculated using a paired t-test. Error bars indicate standard error of mean. $^*p = 0.0261$. (**D**) Western blot showing methylation of purified His-Scd6-Flag in the presence of His-Hmt1 and 1 mM S-adenosyl methionine (SAM).

© The Author(s)
